# Efficacy and safety of avapritinib in advanced systemic mastocytosis: 4-year follow-up of the PATHFINDER study^∗^

**DOI:** 10.1182/bloodadvances.2025017519

**Published:** 2026-01-30

**Authors:** Jason Gotlib, Andreas Reiter, Deepti H. Radia, Iván Álvarez-Twose, Michael W. Deininger, Tracy I. George, Jens Panse, Andrzej Mital, Kristen M. Pettit, Alessandro M. Vannucchi, Uwe Platzbecker, Olivier Hermine, Amro Elshoury, Cristina Bulai Livideanu, Ruben Mesa, Celalettin Ustun, Massimo Triggiani, Ingunn Dybedal, Joseph G. Jurcic, Roberta Zanotti, Stephen T. Oh, Abdulraheem Yacoub, Elizabeth O. Hexner, Prithviraj Bose, Stephanie G. Lee, Wolfgang R. Sperr, Elizabeth A. Griffiths, Matthew Butler, Johannes Lübke, Ilda Bidollari, Hui-Min Lin, Svetlana Rylova, Saša Dimitrijević, Javier I. Muñoz-González, Daniel J. DeAngelo

**Affiliations:** 1 Division of Hematology, Stanford Cancer Institute/Stanford University School of Medicine, Stanford, CA; 2 Department of Hematology and Oncology, University Hospital Mannheim, Heidelberg University, Mannheim, Germany; 3 Guy’s and St Thomas’s NHS Foundation Trust, London, United Kingdom; 4 Institute of Mastocytosis Studies of Castilla-La Mancha, Virgen del Valle Hospital, Toledo, Spain; 5 Department of Internal Medicine, Division of Hematology and Oncology, University of Michigan, Ann Arbor, MI; 6 ARUP Laboratories, Department of Pathology, University of Utah School of Medicine, Salt Lake City, UT; 7 Department of Oncology, Hematology, Hemostaseology and Stem Cell Transplantation, University Hospital RWTH Aachen, Aachen, Germany; 8 Center for Integrated Oncology, Aachen, Germany; 9 Department of Hematology and Transplantology, Medical University of Gdańsk, Gdańsk, Poland; 10 Center for Research and Innovation of Myeloproliferative Neoplasms, Azienda Ospedaliera Universitaria Careggi, University of Florence, Florence, Italy; 11 Department of Hematology, Hemostaseology and Cellular Therapy, University Hospital Leipzig, Leipzig, Germany; 12 Department of Hematology, French National Reference Center for Mastocytosis, Necker-Enfants Malades Hospital, Assistance publique–Hôpitaux de Paris, and Imagine Institute, INSERM U1163, Paris University, Paris, France; 13 Innovative Hematology and Indiana Hemophilia and Thrombosis Center, Indianapolis, IN; 14 Department of Dermatology, Centre of Reference for Mastocytosis, Toulouse University Hospital, Toulouse, France; 15 Atrium Health Wake Forest Baptist Comprehensive Cancer Center, Wake Forest University School of Medicine, Winston Salem, NC; 16 Department of Internal Medicine, Division of Hematology, Oncology and Cell Therapy, Section of Bone Marrow Transplantation and Cellular Therapy, Rush Medical College, Chicago, IL; 17 Division of Allergy and Clinical Immunology, University of Salerno, Salerno, Italy; 18 Departments of Hematology and Pharmacology, Oslo University Hospital, Rikshospitalet, Oslo, Norway; 19 Herbert Irving Comprehensive Cancer Center, Columbia University, New York, NY; 20 Hematology Unit, Department of Medicine, University Hospital of Verona, Verona, Italy; 21 Siteman Cancer Center at Barnes-Jewish Hospital and Washington University, St. Louis, MO; 22 Department of Internal Medicine, The University of Kansas Medical Center, Kansas City, KS; 23 Abramson Cancer Center, Perelman Center for Advanced Medicine, University of Pennsylvania, Philadelphia, PA; 24 Department of Leukemia, The University of Texas MD Anderson Cancer Center, Houston, TX; 25 Department of Medicine, St. Michael’s Hospital, University of Toronto, Toronto, Canada; 26 Department of Medicine, Medical University of Vienna, Vienna, Austria; 27 Leukemia Division, Department of Medicine, Roswell Park Comprehensive Cancer Center, Buffalo, NY; 28 Department of Medicine, Mays Cancer Center, San Antonio, TX; 29 Blueprint Medicines Corporation, Cambridge, MA; 30 Blueprint Medicines (Switzerland) GmbH, Zug, Switzerland; 31 Department of Medical Oncology, Dana-Farber Cancer Institute, Boston, MA

## Abstract

Advanced systemic mastocytosis (AdvSM), a clonal hematologic neoplasm driven predominantly by D816V-mutant *KIT*, is often characterized by organ damage. Associated hematologic neoplasms (AHNs; usually myeloid) are often present, leading to poor survival. We report on the oral, highly selective, potent KIT D816V inhibitor avapritinib (200 mg once daily, starting dose) with >4 years follow-up from the fully enrolled PATHFINDER study. End points included overall response rate (ORR; primary), duration of response (DOR), progression-free survival (PFS), overall survival (OS), changes in objective biomarkers of disease, and safety (all secondary). Of 107 patients with AdvSM (including 71 [66%] with SM-AHN; overall population median follow-up, 49 months), 83 were response-evaluable. ORR was 73% (95% confidence interval, 63-83). Median DOR was 58 months; PFS, 51 months, and OS, 62 months. Disease progression occurred in 21 of 107 patients (20%), predominantly in SM-AHN and largely driven by the AHN. Reductions in objective biomarkers of disease were observed. Most frequent (≥30% patients) treatment-emergent adverse events (TEAEs; any grade; grade ≥3) were thrombocytopenia (58%; 31%); periorbital edema (57%; 6%), anemia (54%; 33%), peripheral edema (48%; 2%), and diarrhea (36%; 5%). Adverse events of special interest were cognitive effects (34%; 8%) and intracranial bleeds (4%; 2%). Eleven (10%) patients experienced TEAEs leading to death, of which 1 was deemed related to avapritinib by the principal investigator. With 4-year follow-up, patients with AdvSM treated with avapritinib experienced deep and durable responses and a favorable benefit-risk profile. This trial was registered at www.ClinicalTrials.gov as #NCT03580655.

## Key Points

• Avapritinib elicited a high rate of deep and durable responses (73% ORR); median OS was 62 months after 4 years follow-up.
• The rate of disease progression during avapritinib treatment was 20% and manifested mostly as progression of the AHN.

## Visual Abstract

**Figure:**
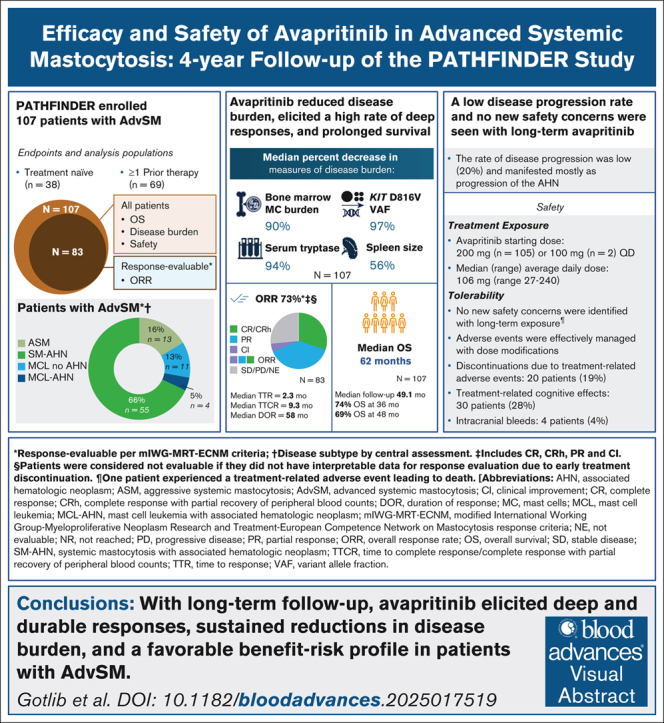

## Introduction

Systemic mastocytosis (SM), including advanced SM (AdvSM), is a rare clonal hematologic neoplasm driven by the *KIT* D816V mutation in ∼95% of cases.^1^, ^2^, ^3^, ^4^ The prevalence of SM has been estimated at up to 1 in 5000 people.^5^, ^6^, ^7^, ^8^ AdvSM is characterized by the accumulation and infiltration of neoplastic *KIT* D816V-mutant mast cells and, potentially, by non–mast cell lineages, which may result in organ damage, historically referred to as C-findings. For example, World Health Organization–defined C-findings include cytopenias; liver function abnormalities (eg, elevated alkaline phosphatase) with or without portal hypertension and ascites; palpable splenomegaly with hypersplenism and/or malabsorption, which may be associated with hypoalbuminemia and weight loss; and large (≥2 cm) lytic bone lesions with or without pathologic factures and/or bone pain. Together with organ damage, mast cell mediator symptoms and cutaneous mast cell lesions (when present) often lead to diminished quality of life and poor survival^1^^,^^9^, ^10^, ^11^, ^12^; indeed, median overall survival (OS) in AdvSM with midostaurin, an approved therapy, has been reported as <2.5 years.^13^

There are 3 recognized subtypes of AdvSM: aggressive SM (ASM), SM with an associated hematologic neoplasm (SM-AHN), and mast cell leukemia (MCL).^14^, ^15^, ^16^ SM-AHN is the most prevalent and accounts for up to 70% of AdvSM diagnoses.^12^^,^^17^ Cells derived from the AHN may also infiltrate various organs, making it a challenge to know the degree to which organ damage is related to the SM or AHN (or both). The most frequently reported AHN subtypes include chronic myelomonocytic leukemia and myelodysplastic/myeloproliferative neoplasms unclassifiable; however, myelodysplastic syndromes, myeloproliferative neoplasms, chronic eosinophilic leukemia, and acute myeloid leukemia (AML) have also been reported.^18^

In AdvSM, gene mutations associated with myeloid malignancies are often observed alongside the *KIT* D816V mutation, resulting in a complex genetic landscape. Of these, *SRSF2*, *ASXL1*, and *RUNX1* (*S/A/R*) are high-risk mutations that have been shown to adversely affect survival.^19^, ^20^, ^21^, ^22^ The mutation-adjusted risk score (MARS) is a validated prognostic score that combines this mutational profile with clinical characteristics to estimate risk and improve treatment stratification.^23^

Avapritinib is a highly potent and selective KIT D816V inhibitor approved by the US Food and Drug Administration for the treatment of AdvSM in adults regardless of previous therapy, and by the European Medicines Agency as monotherapy for the treatment of ASM, SM-AHN, or MCL in adult patients, after ≥1 previous systemic therapies. Avapritinib approval in AdvSM was based on the phase 1 open-label, single-arm, dose-escalation EXPLORER study (ClinicalTrials.gov identifier: NCT02561988) and an interim analysis of 62 enrolled patients in the phase 2 open-label, single-arm PATHFINDER study (NCT03580655),^24^, ^25^, ^26^, ^27^, ^28^ 32 of whom were response-evaluable (median follow-up of 10.4 months) by modified International Working Group–Myeloproliferative Neoplasms Research and Treatment and European Competence Network on Mastocytosis (mIWG-MRT-ECNM) criteria described previously^26^^,^^27^ and shown in supplemental Table 1. In both studies, avapritinib demonstrated a 75% overall response rate (ORR) per mIWG-MRT-ECNM criteria.^26^^,^^27^ Responses were rapid, durable, and observed across AdvSM subtypes regardless of previous therapy.^26^, ^27^, ^28^

Considering the promising results from previous reports, a deeper understanding of the long-term treatment with avapritinib in AdvSM is required. Here, we report the efficacy and safety of avapritinib from the fully enrolled AdvSM population from the pivotal PATHFINDER study with a median follow-up of 4 years.

## Methods

### Study design

PATHFINDER (ClinicalTrials.gov identifier: NCT03580655) is an international, multicenter, open-label, single-arm, phase 2 registrational study evaluating the efficacy and safety of avapritinib in adult patients with centrally confirmed AdvSM conducted in the United States, Canada, and Europe (supplemental Figure 1). Eligible patients received a starting dose of avapritinib 200 mg once a day in 28-day cycles until confirmed progressive disease (PD) of SM (by mIWG-MRT-ECNM criteria); clinical progression of SM or AHN (decided by the investigator to require immediate initiation of cytoreductive therapy); avapritinib was no longer tolerated; patient withdrawal from the study; or death. Dose modification between 25 and 300 mg once a day was permitted according to prespecified criteria. This study was conducted according to the Declaration of Helsinki and good clinical practice guidelines. The protocol was approved by the institutional review board or independent ethics committee of each participating center. All patients provided written informed consent.

Full study design details, including eligibility, outcomes, assessments, and statistical analysis methods have been previously reported.^26^

### Eligibility

Eligible patients (≥18 years of age) had centrally confirmed diagnosis of AdvSM per World Health Organization criteria (2016)^29^ and had an Eastern Cooperative Oncology Group performance status of 0 to 3. Patients were excluded if they had AML or high-risk myelodysplastic syndromes or Philadelphia chromosome–positive malignancies. To mitigate the risk of intracranial bleeds, as seen in earlier studies,^27^ patients with a platelet count of <50 × 10^9^/L were excluded. The mIWG-MRT-ECNM response-evaluable population, as adjudicated by a central committee, included patients with a diagnosis of AdvSM who had received ≥1 dose of avapritinib; ≥2 postbaseline bone marrow assessments and been on study for ≥24 weeks, or had an end-of-study visit; and ≥1 evaluable C-finding (severe and quantifiable organ damage) or MCL regardless of C-findings. Patients with ASM or SM-AHN who did not have an evaluable C-finding were not considered response-evaluable. Evaluable C-findings per mIWG-MRT-ECNM criteria have been described previously.^26^

### Outcomes and assessments

The primary end point was centrally adjudicated ORR by mIWG-MRT-ECNM criteria and included complete remission (CR), CR with partial hematologic recovery (CRh), partial remission (PR), and clinical improvement (CI). Other responses included stable disease (not meeting criteria for CR/CRh, PR, CI, or PD) and PD (as reported by the principal investigators or adjudicated by the study steering committee according to the mIWG-MRT-ECNM criteria; supplemental Table 1).^26^^,^^27^ Select prespecified secondary end points included: time to any response (time from first dose to the time of an initial evaluation of CI or better); duration of response (DOR; time from an initial documented CI or better to the time of an initial documented confirmed PD, loss of response, or death due to any cause, whichever occurs first); progression-free survival (PFS; time from first dose to the time of initial documented confirmed PD or death due to any cause, whichever occurs first); OS (time from first dose to the time of death due to any cause); mean percentage change from baseline in measures of bone marrow mast cell burden, serum tryptase level, blood *KIT* D816V variant allele frequency (VAF), and spleen volume; and safety. Responses according to pure pathologic response (PPR) were also assessed as a prespecified secondary end point and included morphologic CR (absence of bone marrow mast cell aggregates, serum tryptase of <20 ng/mL), morphologic CRh with full or partial hematologic recovery, and morphologic PR (≥50% reduction in bone marrow mast cells and serum tryptase level). To be considered response-evaluable according to PPR, patients had baseline bone marrow mast cell aggregates and/or serum tryptase of ≥20 ng/mL.

For evaluation of bone marrow mast cell burden, formalin-fixed, paraffin-embedded bone marrow biopsies were sectioned at 3- to 4-μm thickness, and stained with hematoxylin and eosin, or underwent immunohistochemistry for CD117, tryptase, CD25, CD30, and CD34, using standard methods. Peripheral blood samples were drawn for serum tryptase and *KIT* D816V VAF analysis, the latter by droplet digital polymerase chain reaction assay with a limit of detection of 0.02%. MARS was conducted according to published criteria and included low- (0-1), intermediate- (2), and high-risk (3-5) groups.^23^

In addition, post hoc logistic regression analyses on predictors of CR/CRh or PR, and Cox regression analyses on predictors of OS were conducted in the mIWG-MRT-ECNM response-evaluable population; these analyses were not prespecified in the study protocol.

The safety population included all enrolled patients from the PATHFINDER study. Treatment-emergent adverse events (TEAEs) were summarized by preferred term (according to the Medical Dictionary for Regulatory Activities version 18.1), severity (graded by the National Cancer Institute Common Terminology Criteria for Adverse Events, version 5.0), seriousness, and relationship to study treatment. A treatment-related AE (TRAE) was defined as any AE likely or possibly related to avapritinib as assessed by the study investigator. Based on the incidence, medical importance, or potential clinical consequences, 2 grouped AEs of special interest were considered in the study: cognitive effects, and intracranial bleeding (ICB). Grouped AE terms included AEs that describe similar medical concepts.

### Statistical methods

Statistical methodology has been described previously.^26^ The database lock was 13 March 2025. All safety analyses and secondary analyses were evaluated in the safety population, comprising patients who received ≥1 dose of avapritinib. Safety analyses were descriptive. For regression analyses, the following variables were assessed: age, diagnosis of ASM vs MCL, diagnosis of SM-AHN vs MCL, AHN presence (yes/no), previous antineoplastic therapy (yes/no), *S/A/R* mutational status, serum tryptase at baseline (as described earlier), bone marrow mast cell burden at baseline (as described earlier), and *KIT* D816V VAF at baseline. For OS regressions only, CR/CRh or PR (yes/no) and *KIT* D816V VAF best response status (positive/negative) were also assessed. All statistical analyses were conducted using SAS version 9.4 or higher.

## Results

### Patients

As of 13 March 2025, there were 107 patients with AdvSM who initiated avapritinib 200 mg (n = 105) or 100 mg (n = 2) once a day (supplemental Figure 1) and had median follow-up of 49.1 months (95% confidence interval [95% CI], 44.2-52.2). The median age was 68 years (range, 31-88), and 45 patients (42%) were female (Table 1). Thirty-eight patients (36%) had never received previous systemic therapy (henceforth termed treatment-naïve), and 69 patients (64%) had received ≥1 previous systemic therapy. The most common subtype was SM-AHN, comprising 71 patients (66%). AHNs were also observed in 4 of 15 patients with MCL.

**Table 1.** **Baseline patient characteristics**

	All AdvSM (N = 107)	Patients with ≥1 previous therapy (n = 69)	Treatment-naïve patients (n = 38)	Response-evaluable AdvSM (n = 83)
Age, median (range), y	68 (31-88)	68 (31-86)	68 (39-88)	68 (31-88)
Female, n (%)	45 (42)	27 (39)	18 (47)	30 (36)
**ECOG performance status, n (%)**				
0-1	79 (74)	48 (70)	31 (82)	60 (72)
2-3	28 (26)	21 (30)	7 (18)	23 (28)
**AdvSM subtype per central assessment, n (%)**				
ASM	21 (20)	14 (20)	7 (18)	13 (16)
SM-AHN	71 (66)	43 (62)	28 (74)	55 (66)
CMML	31 (29)	20 (29)	11 (29)	27 (33)
MDS	12 (11)	6 (9)	6 (16)	6 (7)
MPN	3 (3)	3 (4)	0	2 (2)
MDS/MPN-U	15 (14)	8 (12)	7 (18)	12 (14)
CEL	6 (6)	3 (4)	3 (8)	5 (6)
Other	4 (4)	3 (4)	1 (3)	3 (4)
MCL∗	15 (14)	12 (17)	3 (8)	15 (18)
*KIT* D816V mutation by central assay, n (%)	103 (96)	67 (97)	36 (95)	80 (96)
*KIT* D816V VAF,† median (range), %	16 (ND to 47)	20 (ND to 47)	6 (ND to 45)	19 (ND to 47)
*S/A/R* mutation per central assay,‡ n (%)	48 (45)	25 (36)	23 (61)	40 (48)
**MARS score, n (%)**				
Low	40 (37)	26 (38)	14 (37)	26 (31)
Intermediate	26 (24)	18 (26)	8 (21)	21 (25)
High	41 (38)	25 (36)	16 (42)	36 (43)
BM mast cell burden, median (range), %	40 (1-95)	50 (1-95)	35 (3-90)	50 (1-95)
Serum tryptase level, median (range), ng/mL	262 (24-1600)	312 (24-1600)	178 (37-1336)	312 (24-1600)
Spleen volume, median (range), mL	839 (44-2897)	830 (44-2652)	863 (150-2897)	990 (44-2897)
1 previous systemic therapy, n (%)	42 (39)	42 (61)	0	32 (39)
**Previous antineoplastic therapy, n (%)**				
Midostaurin	58 (54)	58 (84)	0	43 (52)
Cladribine	12 (11)	12 (17)	0	10 (12)
Imatinib	5 (5)	5 (7)	0	5 (6)
Interferon	11 (10)	11 (16)	0	7 (8)

BM, bone marrow; CEL, chronic eosinophilic leukemia; CMML, chronic myelomonocytic leukemia; ddPCR, droplet digital polymerase chain reaction; ECOG, Eastern Cooperative Oncology Group; MDS, myelodysplastic syndrome; MPN, myeloproliferative neoplasms; MPN-U, myeloproliferative neoplasm, unclassifiable; ND, not detected; NGS, next-generation sequencing.

∗ Among patients with the subtype MCL (n = 15), 4 patients had MCL-AHN, and 11 patients had MCL with no AHN.

† Assessed by ddPCR in both the peripheral blood and BM (most were in the peripheral blood; limit of detection, 0.02%).

‡ Assessed by NGS.

At baseline, 103 patients (96%) were positive for the *KIT* D816V mutation, 101 patients (94%) carried ≥1 additional somatic mutation, and ≥1 *S/A/R* mutation was observed in 48 patients (45%). Additional patient demographics and baseline characteristics are summarized in Table 1. A breakdown of additional tier 1 and tier 2 mutations identified using next-generation sequencing is presented in supplemental Table 2. The most common (≥20% of patients) mIWG-MRT-ECNM C-findings at baseline were palpable splenomegaly of ≥5 cm (42%), transfusion-independent anemia (42%), and elevated (grade ≥2) alkaline phosphatase (35%; supplemental Table 3).

### Responses

From the total population (N = 107), 24 patients did not have evaluable C-findings, leaving 83 patients who were response-evaluable according to mIWG-MRT-ECNM criteria, with a median follow-up of 51.8 months (95% CI, 48.0-55.0). As a result of not being response-evaluable per mIWG-MRT-ECNM criteria, these patients were not included in these response analyses. The best confirmed ORR was 73% (n = 61/83; 95% CI, 63-83). Twenty-five patients (30%) achieved CR/CRh, 32 patients (39%) achieved PR, and 4 patients (5%) had CI (Table 2). Responses deepened over time and are shown in supplemental Figure 2. Responses were observed in patients regardless of AdvSM subtype or previous treatment history (Table 2). Best ORR by treatment history according to AdvSM subtype is presented in supplemental Table 4. In patients with a mIWG-MRT-ECNM response (n = 61), the median time to any response was 2.3 months (range, 0.3-20.3) and median time to CR/CRh was 9.3 months (range, 1.8-36.8). Median time to any response and CR/CRh by AdvSM subtype and treatment history is reported in supplemental Table 4. Resolution of C-findings from baseline in the mIWG-MRT-ECNM response-evaluable population with baseline C-findings is shown in supplemental Table 3.

**Table 2.** **Response rates in all response-evaluable patients (mIWG-MRT-ENCM criteria) by AdvSM subtype and treatment history**

Best confirmed response, n (%)	All response-evaluable					
		AdvSM subtype				
	All (n = 83)	ASM (n = 13)	SM-AHN (n = 55)	MCL∗ (n = 15)	Patients with ≥1 previous systemic therapy (n = 53)	Treatment-naïve patients (n = 30)
ORR†	**61 (73)**	10 (77)	41 (75)	10 (67)	**35 (66)**	**26 (87)**
95% CI	**63-83**	46-95	61-85	38-88	**52-79**	**69-96**
**Best response**						
CR/CRh‡	**25 (30)**	4 (31)	18 (33)	3 (20)	**12 (23)**	**13 (43)**
CR	**14 (17)**	1 (8)	10 (18)	3 (20)	**6 (11)**	**8 (27)**
CRh	**11 (13)**	3 (23)	8 (15)	0	**6 (11)**	**5 (17)**
PR§	**32 (39)**	6 (46)	19 (35)	7 (47)	**19 (36)**	**13 (43)**
CI	**4 (5)**	0	4 (7)	0	**4 (8)**	**0**
SD	**13 (16)**	3 (23)	7 (13)	3 (20)	**10 (19)**	**3 (10)**
PD‖	**2 (2)**	0	1 (2)	1 (7)	**2 (4)**	**0**
NE¶	**7 (8)**	0	6 (11)	1 (7)	**6 (11)**	**1 (3)**

Bold includes all subtypes vs the plainfont values which are in individual AdvSM subtypes.

BM, bone marrow; NE, not evaluable; SD, stable disease.

∗ The MCL subtype includes patients with the subtypes MCL (n = 11) and MCL-AHN (n = 4).

† CR + CRh + PR + CI.

‡ CRh requires full resolution of all evaluable C-findings, elimination of BM mast cell aggregates, serum tryptase of <20 ng/mL, resolution of palpable hepatosplenomegaly, and partial hematologic recovery (defined as absolute neutrophil count of >0.5 × 10^9^/L with normal differential, platelet count of >50×10^9^/L, and hemoglobin level of >8.0 g/dL).

§ PR requires full resolution of ≥1 evaluable C-findings and ≥50% reduction in both BM mast cells and serum tryptase.

‖ Two patients had PD as best response.

¶ Patients were considered NE if they did not have interpretable data for response evaluation due to early treatment discontinuation.

Regression analyses of predictors of response are shown in the supplemental Results.

Median DOR in all response-evaluable patients was 58 months (95% CI, 46 to not evaluable), with DOR rates of 74% (95% CI, 63-86) at 36 months and 64% (95% CI, 50-78) at 48 months observed. In treatment-naïve patients, median DOR was not reached (NR), and was 58 months (46 to not evaluable) in patients with ≥1 previous systemic therapy. At 48 months, 62% (95% CI, 40-84) of treatment-naïve patients and 66% (95% CI, 48-84) of patients with ≥1 previous systemic therapy maintained a response to treatment. The DOR in these populations by subtype is shown in supplemental Figure 3.

In the PPR-evaluable population (N = 107), best confirmed ORR per PPR criteria was 74% (79/107; 95% CI, 64-82) and was consistent across AdvSM subtypes. In total, 55 patients (51%) achieved a CR/CRh, and 24 patients (22%) achieved a PR (supplemental Table 5).

### Effect on markers of mast cell disease burden

In the safety population (N = 107), 92 of 105 (88%) patients with baseline and postbaseline assessments had a ≥50% reduction from baseline in bone marrow mast cells, and 78 of 105 (74%) achieved a total clearance of bone marrow mast cell aggregates. Serum tryptase levels were decreased by ≥50% in 98 of 107 patients (92%), with 70 of 107 (65%) achieving a serum tryptase level of <20 ng/mL. In total, 88 of 107 patients (82%) achieved a ≥50% reduction of *KIT* D816V VAF, 68 of 107 patients (64%) achieved a VAF of <1%, and 15 of 107 patients (14%) achieved a VAF reduction under the limit of detection (<0.02%). Spleen volume was reduced by ≥35% in 76 of 105 patients (72%) and in 20 of 33 (61%) with baseline spleen palpation of ≥5 cm. In addition, a nonpalpable spleen was the best response in 42 of 54 patients (78%) who had a palpable spleen at baseline.

**Figure 1.**
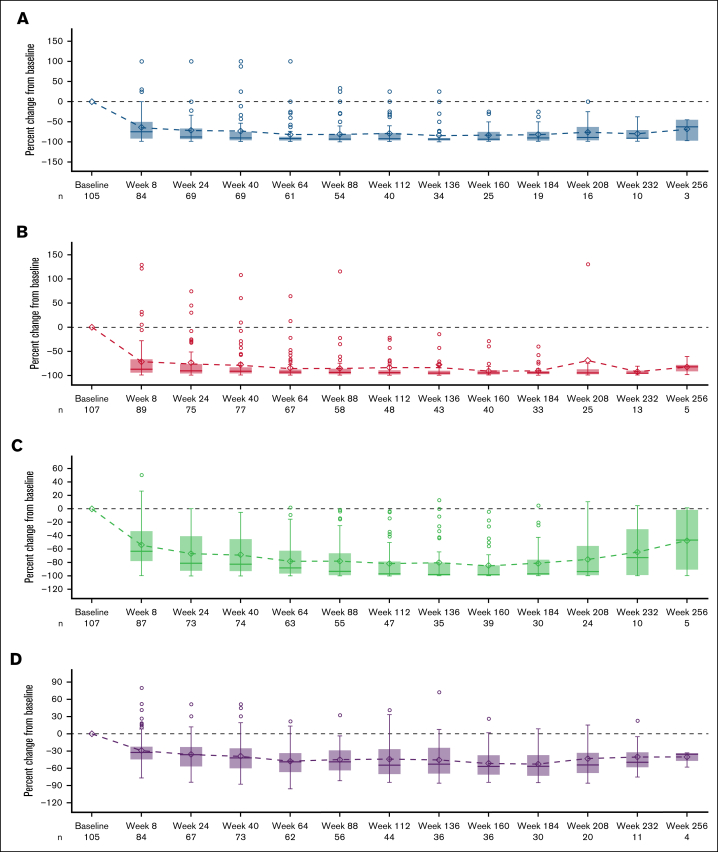
**Mean percentage change from baseline over time in biomarkers of disease.** (A) Bone marrow mast cells. (B) Serum tryptase. (C) *KIT* D816V VAF. (D) Spleen volume. Note: Outliers with >150% change from baseline are not shown (n = 1 for panel A; n = 4 for panel B).

Improvements in objective biomarkers of disease burden were durable and sustained through 4 years of follow-up. Box plots depicting reductions in biomarkers of disease burden over time in AdvSM can be seen in Figure 1. At 48 months, median percentage change from baseline was −90% in bone marrow mast cells, −94% in serum tryptase, −97% in *KIT* D816V VAF, and −56% for spleen volume.

Of 27 patients with monocytosis at baseline, 26 patients (96%) had a ≥50% reduction in monocytes from baseline, and 23 (85%) achieved normalization of monocyte counts (<0.8 × 10^9^/L). There were 23 patients with baseline eosinophilia (absolute eosinophil count of >0.5 × 10^9^/L), with 22 (96%) showing a ≥50% reduction from baseline of absolute eosinophil counts, and all 23 achieving normalization (<0.5 × 10^9^/L).

### Survival measures

**Figure 2.**
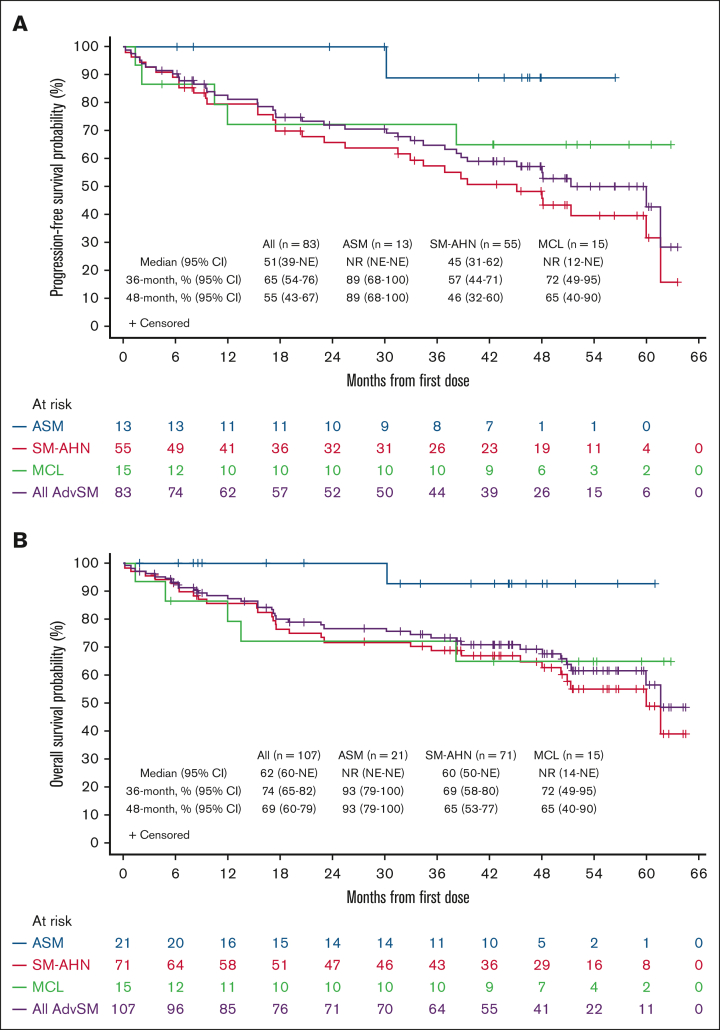
**Kaplan-Meier estimates for PFS and OS by AdvSM subtype.** (A) PFS in all response-evaluable patients. (B) OS in the safety population.

Median PFS in the response-evaluable population (n = 83) was 51 months (95% CI, 39 to not evaluable), 45 months (95% CI, 31-62) in SM-AHN, and NR in ASM or MCL subtypes (Figure 2). Median PFS was NR in treatment-naïve patients and was 48 months (95% CI, 31-62) in patients with ≥1 previous systemic therapy (supplemental Figure 4).

**Figure 3.**
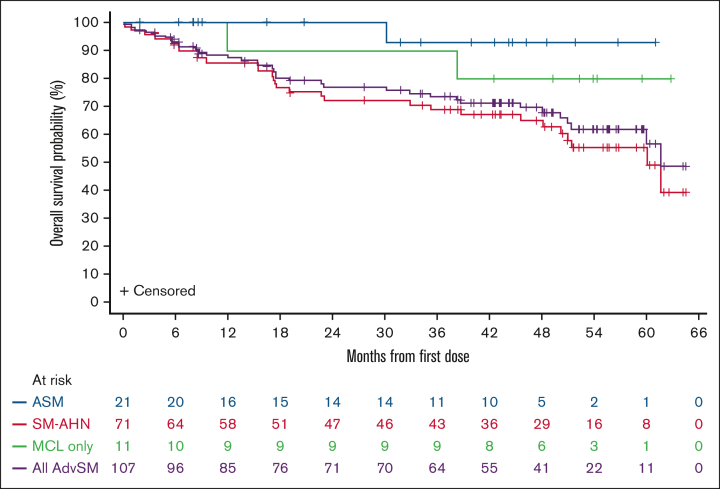
**OS in safety population by AdvSM subtype including MCL with no AHN.**

Median OS in the safety population (N = 107) was 62 months (95% CI, 60 to not evaluable), 60 months (95% CI, 50 to not evaluable) in SM-AHN, and NR in ASM or MCL subtypes (Figures 2 and 3). In 4 patients with MCL-AHN, median OS was 9.2 months (95% CI, 1.4 to not evaluable). In treatment-naïve patients, median OS was NR and was 60 months (95% CI, 48 to not evaluable) in patients with ≥1 previous systemic therapy (supplemental Figure 5). OS was not affected by the number of C-findings at baseline and remained generally consistent in patients with AdvSM when stratified by number of C-findings at baseline (supplemental Figure 6). Based on MARS risk category subgrouping, median OS in patients with low and intermediate scores was NR regardless of history of previous systemic therapy. Patients with a high MARS score (n = 41) had a median OS of 48 months (95% CI, 23 to not evaluable; supplemental Figure 7).

Regression analyses of predictors of OS are shown in the supplemental Results.

### Disease progression

Disease progression was observed in 21 patients, including 6 with progression to AML; 19 of 21 had an initial diagnosis of SM-AHN or MCL-AHN. Patient characteristics for these 21 patients are presented in supplemental Table 6.

### Safety

The median treatment duration of the safety population (N = 107) was 32.7 months (range, 0.2-63.5), with no patients still on treatment at the final database lock due to the end of the study (supplemental Figure 1); all 32 patients remaining on treatment before database lock continued avapritinib treatment outside of PATHFINDER. There were no new safety concerns identified with longer treatment.^26^

All patients experienced ≥1 TEAE. The most frequent (occurring in ≥25%) hematologic TEAEs (any grade; grade ≥3) were thrombocytopenia (58%; 31%), anemia (54%; 33%), and neutropenia (34%; 30%). The most frequent nonhematologic TEAEs (any grade; grade ≥3) were periorbital edema (57%; 6%), peripheral edema (48%; 2%), diarrhea (36%; 5%), nausea (27%; <1%), and arthralgia (26%; 2%). TEAEs and TRAEs (any grade; grade ≥3) occurring in ≥15% of patients are presented in Table 3.

**Table 3.** **TEAEs and TRAEs of safety population (N = 107)**

	Any-cause TEAEs∗		TRAEs	
	Any grade	Grade ≥3	Any grade	Grade ≥3
Any, n (%)	107 (100)	95 (89)	103 (96)	73 (68)
**Nonhematologic AEs in ≥15%, n (%)**				
Periorbital edema†	61 (57)	6 (6)	59 (55)	6 (6)
Peripheral edema†	51 (48)	2 (2)	42 (39)	2 (2)
Diarrhea	38 (36)	5 (5)	16 (15)	1 (<1)
Nausea	29 (27)	1 (<1)	13 (12)	0
Arthralgia	28 (26)	2 (2)	7 (7)	0
Vomiting	25 (23)	2 (2)	9 (8)	1 (<1)
Face edema†	24 (22)	0	23 (21)	0
Fatigue	24 (22)	3 (3)	14 (13)	2 (2)
COVID-19	22 (21)	4 (4)	0	0
Epistaxis	20 (19)	0	6 (6)	0
Pruritus	19 (18)	0	5 (5)	0
Hair color changes	18 (17)	0	18 (17)	0
Rash	18 (17)	1 (<1)	7 (7)	1 (<1)
Blood alkaline phosphatase increased	17 (16)	3 (3)	9 (8)	2 (2)
Abdominal pain	16 (15)	1 (<1)	2 (2)	0
Blood creatinine increased	16 (15)	1 (<1)	3 (3)	0
Constipation	16 (15)	1 (<1)	4 (4)	0
Headache	16 (15)	0	8 (7)	0
**Hematologic AEs in ≥15%, n (%)**				
Thrombocytopenia†	62 (58)	33 (31)	55 (51)	31 (29)
Anemia†	58 (54)	35 (33)	35 (33)	17 (16)
Neutropenia†	36 (34)	32 (30)	30 (28)	27 (25)
**AEs of special interest in ≥1%, n (%)**				
Cognitive effects†	36 (34)	9 (8)	30 (28)	6 (6)
Cognitive disorder	21 (20)	5 (5)	19 (18)	5 (5)
Memory impairment	10 (9)	0	9 (8)	0
Confusional state	7 (7)	2 (2)	2 (2)	0
Delirium	3 (3)	2 (2)	0	0
ICB†	4 (4)	2 (2)	4 (4)	2 (2)
Subdural hematoma	2 (2)	2 (2)	2 (2)	2 (2)
Intracranial hemorrhage	2 (2)	0	2 (2)	0
AEs leading to death, n (%)	11 (10) ‡		1 (<1)§	

∗ TEAEs were defined as any AE that occurred between the first dose of avapritinib through 30 days after the last dose of avapritinib.

† Pooled terms.

‡ Ten deaths considered not related to treatment included *Escherichia* sepsis, Fournier gangrene, pneumonia aspiration, septic endocarditis, septic shock, erosive gastritis, intra-abdominal hemorrhage, acute kidney injury, cardiac failure, and disease progression.

§ One patient died due to acute kidney injury that was considered related to treatment, an 80-year-old man with a medical history of reduced kidney function and increased blood creatinine who died due to acute kidney injury in the context of treatment with antibiotics due to bilateral pneumonia complicated with diarrhea and vomiting.

Serious AEs occurred in 69 patients (64%) with treatment-related serious AEs occurring in 15 patients (14%; supplemental Table 7). Eleven patients (10%) experienced AEs resulting in death (Table 3), with 1 assessed as related to avapritinib by the investigator. This was an 80-year-old man with a medical history of reduced kidney function and increased blood creatinine who died due to acute kidney injury in the context of treatment with antibiotics for bilateral pneumonia complicated by diarrhea and vomiting.

Edema TEAEs were mostly grade 1 or 2 and managed through dose modification with only 1 patient discontinuing treatment due to peripheral edema.

Cognitive effects and ICBs were events of special interest in the study. Cognitive effects occurred in 34% of patients and were considered treatment-related in 28% of patients. Most cognitive effects were grade 1 or 2, with the most frequent (≥5% of patients; any grade; grade ≥3) being cognitive disorder (20%; 5%), memory impairment (9%; 0%), and confusional state (7%; 2%). Median time to onset for any grade, and improvement and resolution for grade ≥2 cognitive effects are presented in supplemental Table 8. Cognitive effects were generally managed through dose modification. As a result, treatment discontinuations due to cognitive effects were limited and occurred in 4 patients overall; 3 with cognitive disorder, age ranging from 66 to 70 years (2 grade 3, and 1 grade 2) assessed as related to treatment with avapritinib, and 1 with grade 3 dementia in a 71-year-old man, attributed to retrospectively confirmed Alzheimer disease and not related to treatment with avapritinib.

Analyses of ICBs in patients from previous reports of the EXPLORER and PATHFINDER studies identified severe thrombocytopenia at baseline as a significant risk factor for ICBs.^26^^,^^27^ Consequently, patients with baseline platelet counts of <50 × 10^9^/L were excluded from enrollment. In addition, increased platelet count monitoring with guidance for treatment interruption, dose reduction, and support for severe thrombocytopenia or treatment discontinuation were also implemented. These risk mitigation strategies substantially reduced the rate of ICBs in PATHFINDER compared with EXPLORER.^27^ ICBs occurred in 4 patients (4%) enrolled in PATHFINDER and included 2 patients with intracranial hemorrhages and 2 with subdural hematomas. All 4 patients had confounding factors such as medical history of hypertension (n = 3), use of antithrombotic treatment (n = 3), or head trauma around the time the bleeding occurred (n = 1). In addition, 3 of 4 patients experienced severe thrombocytopenia over the course of the study treatment leading to avapritinib dose modification in all 3 patients, platelet transfusions in 2 patients, and treatment with a thrombopoietin receptor agonist in 1 patient. All 4 patients discontinued avapritinib treatment and events were reported to have resolved; none of the patients died due to ICB. Treatment details for patients who had ICBs are presented in supplemental Table 9.

Overall, 38 patients (36%) discontinued treatment due to TEAEs, including 20 patients (19%) due to TRAEs. Dose modifications due to AEs (any-cause; treatment-related) including reductions (79%; 78%), and interruptions (73%; 64%) are reported in supplemental Tables 10 and 11. Dose modifications due to TRAEs (interruptions; reductions) were mostly due to thrombocytopenia (25%; 30%), and neutropenia (21%; 21%).

The median time to first dose reduction and the median average daily dose were 1.6 months (range, 0.0-34.2) and 106 mg (range, 27-240), respectively (supplemental Table 12); of 29 patients who reached 48 months of treatment, 3 were receiving avapritinib >100 mg once a day, 12 were receiving 100 mg once a day, and 14 were receiving <100 mg once a day at that time point. A breakdown of patient dosage over time is shown in supplemental Table 13.

## Discussion

To our knowledge, this is the largest and longest prospective interventional study of any highly selective KIT inhibitor in AdvSM. The results of the PATHFINDER study provide important guidance and support for the use of avapritinib in clinical practice. With 4 years of follow-up, median OS and PFS were 62 months and 51 months, respectively, reflecting a high rate of deep (73% median ORR; 30% CR/CRh) and durable (median DOR of 58 months) responses, as accompanied by persistent reductions in all disease burden biomarkers. A low rate of disease progressions was observed, primarily in patients with SM-AHN, and manifested mostly as progression of the AHN. Avapritinib dose modifications were generally effective in the management of AEs including cytopenias, edema, and cognitive effects, allowing patients to remain on avapritinib therapy.

The deep and durable responses and OS in patients compare favorably with results observed with other AdvSM therapies. The multikinase/KIT inhibitor midostaurin demonstrated a median OS of 28.7 months and median DOR of 24.1 months in AdvSM.^13^ In a retrospective external control study, avapritinib-treated patients from PATHFINDER and EXPLORER studies had significantly superior OS compared with patients receiving best available therapy (mainly midostaurin and cladribine).^30^ In a more recent retrospective analysis comparing PATHFINDER and EXPLORER results specifically with those of midostaurin or cladribine, avapritinib treatment resulted in significantly improved OS, longer duration of treatment, and greater reduction in serum tryptase levels compared with midostaurin or cladribine in real-world clinical practice.^31^ Furthermore, the effectiveness of avapritinib extended across all MARS-defined risk groups, and across all AdvSM subtypes, including settings in which other therapies can have diminished efficacy. Although median OS with avapritinib was of shorter duration in the high MARS group vs low and intermediate MARS groups, outcomes were still improved in patients with high MARS with avapritinib in PATHFINDER (median OS of 4.0 years) compared with median OS of 1.2 years with midostaurin in a similar high MARS subgroup.^32^ Similarly, in PATHFINDER, median OS was NR in the MCL subtype, whereas OS reported for midostaurin in a pivotal clinical trial and a registry study were 9.4 months and 2.3 years, respectively.^13^^,^^33^ It should be noted that, in this study, median OS was numerically longer in patients with MCL only than in the small population of patients with MCL-AHN (n = 4). Additionally, based on multivariate logistic regression analysis, treatment-naïve patients were significantly more likely to achieve CR/CRh with avapritinib than patients with a history of previous antineoplastic therapy. In the absence of a randomized controlled clinical trial, results of the PATHFINDER study, supported by published reports, favor avapritinib over other available therapies for adult patients with AdvSM.

The use of PPR criteria provides an option for objective evaluation of response to treatment in a broader patient population, which is not affected by challenges and subjective evaluation errors associated with assessment of C-findings. Rates of CR/CRh were 51% and 30% with PPR and mIWG-MRT-ENCM criteria, respectively, with high ORRs by both criteria (74% and 73%, respectively); PPR responses to avapritinib were similar to previous reports.^34^ The practicality of evaluating only bone marrow mast cell burden, tryptase, and complete blood counts, without the need for C-finding adjudication, makes PPR a useful tool for both community and academic physicians.

This 4-year follow-up of patients treated with avapritinib also provided important insights into the patterns of disease progression, with most progressions reported in patients with the SM-AHN subtype, 1 patient with MCL, and 1 patient with ASM. In addition, progressions were largely driven by the AHN. These observations may support the hypothesis that the AHN either represents a clonally distinct *KIT* D816V–negative neoplasm or a multimutated myeloid malignancy with other non–*KIT* D81V mutations. Nonetheless, avapritinib treatment also resulted in marked decreases of baseline monocytosis and eosinophilia, suggesting its effects may, in part, be due to the presence of *KIT* D816V as a disease driver across different cell lineages of AdvSM and/or to indirect effects, such as reduced cytokine levels.^35^ This highlights a need for the deeper understanding of the clonal dynamics during avapritinib therapy; indeed, in clonal architecture mapping in 4 avapritinib-treated patients with SM-AHN, avapritinib strongly affected the mast cell component but had varying impact on abnormal myeloid cells depending on the cellular distribution of *KIT* D816V and its relative role with comutations in the genomic landscape of such cases.^36^ This also demonstrates the need to evaluate combination treatment strategies with selective KIT D816V–targeting agents.

Finally, the deep and durable responses, particularly the high rates of CR/CRh, reported here, provide an opportunity to reevaluate the role of allogeneic hematopoietic stem cell transplantation (allo-HSCT) in the modern KIT inhibitor era,^37^, ^38^, ^39^ albeit, candidates for allo-HSCT for SM treatment were excluded from this study. For example, patients with ASM or MCL without an AHN demonstrated the highest rates of durable OS on avapritinib and may not require allo-HSCT; in contrast, patients with SM-AHN wherein the AHN component would otherwise be transplanted should be evaluated for allo-HSCT. Further data are needed to understand the potential cytoreductive role of avapritinib before allo-HSCT as well as its potential for preventing or treating posttransplant relapse.

After 4 years of follow-up, no new safety concerns were identified. Patients benefited from a starting dose at 200 mg once a day to enable disease control, although the average daily dose was 106 mg due to dose reductions over time, leading to 3 important conclusions: a flexible avapritinib dosing approach provided treatment benefit to patients with AdvSM, with higher doses used early in treatment to achieve rapid responses; responses were durable and maintained even at lower doses; and dose reductions, when needed, were an effective way to mitigate toxicities, maintaining the favorable safety profile and response to treatment. Indeed, cognitive effects were well managed with dose reductions, with very few leading to discontinuation.

This 4-year follow-up demonstrated that the strategy for the mitigation of ICB risk was effective. Through limiting enrollment to patients with platelet counts of >50 × 10^9^/L, holding treatment in patients with platelet counts of <50 × 10^9^/L, supporting severe thrombocytopenia with platelet transfusions and thrombopoietin receptor agonists (at investigator discretion), and avoiding anticoagulants, the rate of ICBs was substantially reduced compared with previous studies.^27^ There were no fatal occurrences of ICBs reported in this study.

The main limitations of this study include the open-label, uncontrolled design, precluding direct comparison with other therapies, as well as the relatively small study size that, although large for AdvSM, complicates subgroup analyses in this very heterogenous disease.

In conclusion, in this analysis of the PATHFINDER study with a median follow-up of 4 years, patients treated with avapritinib exhibited high rates of CR/CRh, which were similarly associated with high rates of PFS and OS, particularly in patients without an AHN. These outcomes were associated with substantial and sustained reductions in objective biomarkers of disease burden such as bone marrow mast cells, serum tryptase, and *KIT* D816V VAF, as well as reversion of organ damage. Logistic regression analyses also showed that treatment-naïve patients were more likely to achieve complete disease remissions, whereas achievement of CR/CRh and PR was predictive of longer OS. With prolonged follow-up, the well-characterized safety profile of avapritinib remained consistent with that of previous reports, supporting that administration of avapritinib over an extended period maintains a favorable benefit-risk profile in patients with AdvSM.

Conflict-of-interest disclosure: J.G. is the chair of the response adjudication committee for, has received research funding from, served on advisory boards for, and received honoraria and funding to cover travel expenses from, Blueprint Medicines Corporation, a wholly owned subsidiary of Sanofi. A.R. has received research funding, served on advisory boards, and received honoraria and funding to cover travel expenses from AbbVie, AOP Orphan Pharmaceuticals, Blueprint Medicines Corporation, a wholly owned subsidiary of Sanofi, Bristol Myers Squibb (BMS), Cogent, GSK, Incyte, and Novartis. D.H.R. has been a clinical advisory board/study steering group member (EXPLORER/PATHFINDER) for Blueprint Medicines Corporation, a wholly owned subsidiary of Sanofi; has been a study steering committee member for Cogent Biosciences; has been involved in educational events and advisory boards for Novartis; and has received author fees for Medscape cases. I.A.-T. has received research funding from Blueprint Medicines Corporation, a wholly owned subsidiary of Sanofi; and has received advisory board or speaker fees from Blueprint Medicines Corporation, a wholly owned subsidiary of Sanofi and Novartis. M.W.D. has received honoraria fees from Blueprint Medicines Corporation, a wholly owned subsidiary of Sanofi, Incyte, Medscape, Sangamo, and Takeda; received consultancy fees from Blueprint Medicines Corporation, a wholly owned subsidiary of Sanofi, DisperSol, Fusion Pharma, Novartis, and Sangamo; received research funding from Blueprint Medicines Corporation, a wholly owned subsidiary of Sanofi, Incyte, Leukemia & Lymphoma Society, Novartis, Pfizer, Sun Pharma Advanced Research Company, and Takeda; is part of a study management committee for Blueprint Medicines Corporation, a wholly owned subsidiary of Sanofi and Takeda; and is a case author for Medscape. T.I.G. has received consulting fees from, and is a study steering committee member for, Blueprint Medicines Corporation, a wholly owned subsidiary of Sanofi, BMS/Celgene, Cogent Biosciences, and Incyte. J.P. has received honorarium fees from Apellis, Alexion, AstraZeneca, Blueprint Medicines Corporation, a wholly owned subsidiary of Sanofi, BMS, Cogent, F. Hoffmann-La Roche, Grünenthal, Merck Sharp and Dohme, Novartis, Omeros, Samsung Bioepis, and Sobi; has served on the speakers bureau of Alexion, Boehringer Ingelheim, Chugai, Novartis, Pfizer, and Sobi; and is a study steering committee member for Blueprint Medicines Corporation, a wholly owned subsidiary of Sanofi and Omeros. A.M. has received honoraria from Takeda, Pfizer, Novo Nordisk, Behring, AbbVie, Novartis, Cilag, Janssen, and Bayer. K.M.P. has participated in advisory boards for CTI BioPharma, AbbVie, Protagonist Therapeutics, PharmaEssentia, and Kura Oncology. A.M.V. serves on advisory boards for, and has received fees for lectures from, Incyte, Novartis, GSK, AOP Orphan Pharmaceuticals, and Blueprint Medicines Corporation, a wholly owned subsidiary of Sanofi. U.P. received research funding and honoraria from Amgen, Janssen, Jazz, and Novartis. O.H. received research funding from AB Science, BMS-Celgene, Alexion, Novartis, and Inatherys; has consulted for AB Science; and is a shareholder for AB Science. A.E. received compensation for his participation on the speakers bureau of Blueprint Medicines Corporation, a wholly owned subsidiary of Sanofi. C.B.L. has received consultancy fees from Lilly, Novartis, and UCB; has received research support from Novartis; and is a nonpaying member of the steering committee for an AB Science study. R.M. has been a consultant for Novartis, Sobi, GSK, BMS, Incyte, and PharmaEssentia; has received research support from Celgene, Incyte, AbbVie, Samus, Genetech, Promedior, and CTI BioPharma. C.U. reports compensation for their role on the speakers bureau from Blueprint Medicines Corporation, a wholly owned subsidiary of Sanofi and Takeda; serves on the advisory board for Cogent; and holds a nonpaid role for the Mastocytosis Society. M.T. has received fees for advisory boards from Blueprint Medicines Corporation, a wholly owned subsidiary of Sanofi, Deciphera, and Novartis. I.D. has received advisory board fees from Novartis and Blueprint Medicines Corporation, a wholly owned subsidiary of Sanofi. J.G.J. receives research funding paid to Columbia University from Blueprint Medicines Corporation, a wholly owned subsidiary of Sanofi, BMS, Forma Therapeutics, Gilead/Forty Seven, Seagen/Pfizer, and Sumitomo Pharma; serves on advisory boards for Gallop Oncology and Syros Pharmaceuticals; and serves as consultant for Incyte and Rigel Pharmaceuticals. R.Z. reports compensation from Blueprint Medicines Corporation, a wholly owned subsidiary of Sanofi for meeting participation; and has acted as consultant for Istituto Gentili. S.T.O. has been a consultant for Disc Medicine, Blueprint Medicines Corporation, a wholly owned subsidiary of Sanofi, AbbVie, Constellation, CTI BioPharma, BMS, Geron, Sierra Oncology, Cogent, and Incyte. A.Y. has served on advisory boards for Incyte, CTI BioPharma, PharmaEssentia, Pfizer, Novartis, Acceleron Pharma, Servier, AbbVie, Apellis, Gilead, Notable Labs, and Celgene. E.O.H. has received research support (to institution) from Blueprint Medicines Corporation, a wholly owned subsidiary of Sanofi, Disc Medicines, and AbbVie Oncology; serves on a data safety monitoring committee for Blueprint Medicines Corporation, a wholly owned subsidiary of Sanofi; and is a member of the hematology examination committee for the American Board of Internal Medicine. P.B. has received research support from Blueprint Medicines Corporation, a wholly owned subsidiary of Sanofi, Celgene (now BMS), Constellation (now MorphoSys), Cogent, CTI BioPharma (now Sobi), Incyte, Ionis, Kartos, Telios, Disc Medicine, Geron, Janssen, and Sumitomo Oncology Pharma; and has received honoraria from AbbVie, Blueprint Medicines Corporation, a wholly owned subsidiary of Sanofi, Celgene (now BMS), Cogent, Constellation (now MorphoSys), CTI BioPharma (now Sobi), Incyte, Karyopharm, Sumitomo, Morphic, Jubilant, Novartis, PharmaEssentia, and GSK. S.G.L. has received honoraria from Medison; has served on advisory boards for Medison, Novartis, and Forus Therapeutics Inc. W.R.S. has received honoraria, scientific grants, or travel support from AbbVie, Astellas, Blueprint Medicines Corporation, a wholly owned subsidiary of Sanofi, BMS-Celgene, Daiichi Sankyo, Deciphera, Incyte, Jazz, Laboratoires Delbert, Novartis, Otsuka, Pfizer, Servier, Stemline, and Thermo Fisher. E.A.G. has received honoraria with direct payments from The Aplastic Anemia and MDS International Foundation, American Society of Hematology, MDS International Foundation, MediCom Worldwide, Physician Educational Resource, Picnic Health, and WebMD; reports consulting or advisory board participation with direct payments/in kind contributions from AbbVie, Alexion Pharmaceuticals, AstraZeneca Rare Disease, Genentech, Novartis, Apellis, Celgene/BMS, Servier Pharmaceuticals, Takeda Oncology, and Taiho Oncology; and reports research funding to Roswell Park Comprehensive Cancer Center from Alexion Pharmaceuticals, Astex Pharmaceuticals, Blueprint Medicines Corporation, a wholly owned subsidiary of Sanofi, Celldex Therapeutics, Celgene/BMS, Genentech Inc, and NextCure Therapeutics. J.L has received honoraria from Blueprint Medicines Corporation, a wholly owned subsidiary of Sanofi. I.B., H.-M.L., S.R., S.D., and J.I.M.-G. are employees of Blueprint Medicines Corporation, a wholly owned subsidiary of Sanofi. D.J.D. has served as a consultant for Amgen, Autolus, Blueprint Medicines Corporation, a wholly owned subsidiary of Sanofi, Gilead, Kite, Jazz, Novartis, Pfizer, Servier, and Takeda; and received research funding from AbbVie, Blueprint Medicines Corporation, a wholly owned subsidiary of Sanofi, GlycoMimetics, and Novartis. M.B. declares no competing financial interests.
