# Supplementary material for: Efficacy and safety of avapritinib in advanced systemic mastocytosis: 4-year follow-up of the PATHFINDER study
Source: Blood Adv. 2026 Jan 30;10(10):3676–89. doi: 10.1182/bloodadvances.2025017519 (PMC13207509; doi:10.1182/bloodadvances.2025017519)
Supplement: Supplemental Methods, Tables, and Figures [file BLOODA_ADV-2025-017519-mmc1.pdf]

Supplementary Material for:

Efficacy and safety of avapritinib in advanced systemic mastocytosis: 4-year follow-up of the PATHFINDER study

Jason Gotlib,[1,a] Andreas Reiter,[2,a] Deepti H. Radia,[3] Iván Álvarez-Twose,[4] Michael W. Deininger,[5] Tracy I. George,[6] Jens Panse,[7,8] Andrzej Mital,[9] Kristen M. Pettit,[5] Alessandro M. Vannucchi,[10] Uwe Platzbecker,[11] Olivier Hermine,[12] Amro Elshoury,[13] Cristina Bulai Livideanu,[14] Ruben Mesa,[15] Celalettin Ustun,[16] Massimo Triggiani,[17] Ingunn Dybedal,[18] Joseph G. Jurcic,[19] Roberta Zanotti,[20] Stephen T. Oh,[21] Abdulraheem Yacoub,[22] Elizabeth O. Hexner,[23] Prithviraj Bose,[24] Stephanie G. Lee,[25] Wolfgang R. Sperr,[26] Elizabeth A. Griffiths,[27] Matthew Butler,[28] Johannes Lübke,[2] Ilda Bidollari,[29] Hui-Min Lin,[29] Svetlana Rylova,[30] Saša Dimitrijević,[30] Javier I. Muñoz-González,[30] and Daniel J. DeAngelo[31]

[a]Equally contributing authors

[1]Division of Hematology, Stanford Cancer Institute/Stanford University School of Medicine, Stanford, CA;

[2]Department of Hematology and Oncology, University Hospital Mannheim, Heidelberg University, Mannheim, Germany;

[3]Guy's & St Thomas's NHS Foundation Trust, London, United Kingdom;

[4]Institute of Mastocytosis Studies of Castilla-La Mancha, Virgen del Valle Hospital, Toledo, Spain;

[5] Department of Internal Medicine, Division of Hematology and Oncology, University of Michigan Ann Arbor, MI;

[6]ARUP Laboratories, Department of Pathology, University of Utah School of Medicine, Salt Lake City, UT;

[7]*Department of Oncology, Hematology, Hemostaseology and Stem Cell Transplantation, University Hospital RWTH Aachen, Aachen, Germany;*
[8]*Center for Integrated Oncology (CIO), Aachen, Germany;*
[9]*Department of Hematology and Transplantology, Medical University of Gdańsk, Gdańsk, Poland;*
[10]*Center for Research and Innovation of Myeloproliferative Neoplasms (CRIMM), Azienda Ospedaliera Universitaria Careggi, University of Florence, Florence, Italy;*
[11]*Leipzig University, Leipzig, Germany;*
[12]*Department of Hematology, French National Reference Center for Mastocytosis, Necker–Enfants Malades Hospital, Assistance publique–Hôpitaux de Paris, and Imagine Institute, INSERM U1163, Paris University, Paris, France;*
[13]*Innovative Hematology and the Indiana Hemophilia and Thrombosis Center, Indianapolis, IN;*
[14]*Department of Dermatology, Centre of Reference for Mastocytosis, Toulouse University Hospital, Toulouse, France;*
[15]*Atrium Health Wake Forest Baptist Comprehensive Cancer Center, Wake Forest University School of Medicine, Winston Salem, NC;*
[16]*Department of Internal Medicine, Division of Hematology, Oncology and Cell Therapy, Section of Bone Marrow Transplantation and Cellular Therapy, Rush Medical College, Chicago, IL;*
[17]*Division of Allergy and Clinical Immunology, University of Salerno, Salerno, Italy;*
[18]*Departments of Hematology and Pharmacology, Oslo University Hospital, Rikshospitalet, Oslo, Norway;*
[19]*Herbert Irving Comprehensive Cancer Center, Columbia University, New York, NY;*
[20]*Hematology Unit, Department of Medicine, University Hospital of Verona, Verona, Italy;*
[21]*Siteman Cancer Center at Barnes-Jewish Hospital and Washington University, Saint Louis, MO;*
[22]*Department of Internal Medicine, The University of Kansas Medical Center, Kansas City, KS;*
[23]*Abramson Cancer Center, Perelman Center for Advanced Medicine, University of Pennsylvania, Philadelphia, PA;*
[24]*Department of Leukemia, The University of Texas MD Anderson Cancer Center, Houston, TX;*
[25]*Department of Medicine, St. Michael's Hospital, University of Toronto, Toronto, Canada;*
[26]*Department of Medicine, Medical University of Vienna, Vienna, Austria;*
[27]*Leukemia Division, Department of Medicine, Roswell Park Comprehensive Cancer Center, Buffalo, NY;*

[28]*Department of Medicine, Mays Cancer Center, San Antonio, TX;*
[29]*Blueprint Medicines Corporation, Cambridge, MA;*
[30]*Blueprint Medicines (Switzerland) GmbH, Zug, Switzerland;*
[31]*Department of Medical Oncology, Dana-Farber Cancer Institute, Boston, MA*

Supplement

Supplementary Results

Regression analyses of predictors of response and survival

In the univariate and multivariate logistic regression analysis, being treatment-naive was a significant predictor of achieving CR/CRh or PR (**supplemental Table 14**). In the univariate Cox regression analysis, being treatment-naive (HR = 0.35; 95% CI, 0.13-0.92; *P* = .03), CR/CRh or PR (HR = 0.12; 95% CI, 0.05-0.28; *P* < .0001), CR/CRh or PR or CI (HR = 0.12; 95% CI, 0.06-0.28; *P* < .0001), and no AHN (HR = 0.23; 95% CI, 0.07-0.75; *P* = .01) were significantly associated with longer OS. In the multivariate Cox regression analysis, younger age (HR = 1.08; 95% CI, 1.02-1.14; *P* = .008), being treatment naive (HR = 0.25; 95% CI, 0.07-0.95l *P* = 0.04),and CR/CRh or PR (HR = 0.09; 95% CI, 0.02-0.21; *P* < .0001) were significantly associated with longer OS (**supplemental Table 15**).

Supplement Table 1. mIWG-MRT-ECNM criteria for responses in patients with AdvSM

Response	mIWG-MRT-ECNM criteria
Complete remission*<br>(CR)	Requires all 4 of the following criteria, and response duration must be ≥12 weeks:<br>• No presence of compact neoplastic mast cell aggregates in the BM or other biopsied extracutaneous organ<br>• Serum tryptase level <20 ng/mL[†]<br>• Peripheral blood count remission defined as:<br>– ANC ≥1 × 10^9/L with normal differential (absence of neoplastic mast cells and blasts <1%) *and*<br>– Platelet count ≥100 × 10^9/L *and*<br>– Hgb level ≥11 g/dL<br>• Complete resolution of palpable hepatosplenomegaly and all biopsy-proven or suspected SM-related organ damage (C-findings)[‡]
CR with partial recovery of peripheral blood counts*<br>(CRh)	Requires all criteria for CR be met and response duration must be ≥12 weeks; however, patient may have residual cytopenias. The following minimum recovery of peripheral blood counts is required:<br>• ANC >0.5 × 10^9/L with normal differential (absence of neoplastic mast cells and blasts <1%) *and*<br>• Platelet count >50 × 10^9/L *and*<br>• Hgb level >8.0 g/dL
Partial remission*<br>(PR)	Requires all 3 of the following criteria, and response duration must be ≥12 weeks, in the absence of both CR and PD:<br>• Reduction by ≥50% in neoplastic mast cells in the BM and/or other extracutaneous organ at biopsy demonstrating eligible SM-related organ damage<br>• Reduction of serum tryptase level by ≥50%[†]

<table>
<tr><td></td><td colspan="2">Resolution of ≥1 biopsy-proven or suspected SM-related organ damage (C-finding[s])[‡]</td></tr>
<tr><td>Clinical improvement*
(CI)</td><td colspan="2">Response duration must be ≥12 weeks

Requires 1 or more of the nonhematologic and/or hematologic response criteria to be fulfilled in the absence of CR/CRh, PR, or PD</td></tr>
<tr><td>Stable disease
(SD)</td><td colspan="2">Not meeting criteria for CR/CRh, PR, CI, or PD</td></tr>
<tr><td rowspan="7">Progressive disease[‖]
(PD)</td><td colspan="2">Requires at least 1 element from the criteria below; duration must be ≥4 weeks:</td></tr>
<tr><td>Baseline</td><td>≥ Post baseline</td></tr>
<tr><td>Any Grade 2 non-hematologic organ damage</td><td>Worsening by 1 grade and

Minimum 100% increase (doubling) of laboratory abnormality</td></tr>
<tr><td>≥Grade 2 albumin</td><td>Worsening by 1 grade and

Decrease by ≥0.5 g/dL</td></tr>
<tr><td>≥Grade 3 non-hematologic organ damage</td><td>Minimum 100% increase (doubling) of laboratory abnormality</td></tr>
<tr><td>≥Grade 2 transfusion-independent anemia or thrombocytopenia</td><td>New transfusion dependence at 8 weeks of ≥4 units of RBCs or platelets</td></tr>
<tr><td>Transfusion-dependent anemia or thrombocytopenia</td><td>≥100% increase in the average transfusion frequency for an 8-week period compared with the 12 weeks preceding treatment</td></tr>
</table>

	≥Grade 3 neutropenia	>50% decrease in neutrophil count *and*<br><br>Absolute decrease of neutrophil count of ≥0.25 × 10^9/L *and* Grade 4 (<0.5 × 10^9/L)
	Baseline spleen size of not palpable or ≤5 cm	Development of ≥10 cm palpable symptomatic splenomegaly *or*<br><br>Increase in spleen volume ≥25%
	Splenomegaly >5 cm	>50% worsening *and*<br><br>Development of ≥10 cm of palpable symptomatic splenomegaly compared with the baseline value *or*<br><br>Increase in spleen volume ≥25%
Loss of response	Loss of a documented CR/CRh, PR, or CI that must be for ≥8 weeks. Downgrading of CR/CRh to PR, or PR to CI is considered as such but is not considered a loss of response unless CI is also lost for ≥8 weeks. The baseline value for LOR is the pretreatment measurement(s) and not the nadir values during response.	

AdvSM, advanced systemic mastocytosis; ANC, absolute neutrophil count; BM, bone marrow; CI, clinical improvement; CR, complete remission; CRh, complete remission with partial recovery of peripheral blood counts; Hgb, hemoglobin; IWG-MRT-ECNM, International Working Group-Myeloproliferative Neoplasms Research and Treatment and European Competence Network on Mastocytosis; LOR, loss of response; PD, progressive disease; PR, partial remission; RBCs, red blood cells; SD, stable disease; SM, systemic mastocytosis.

Guidelines for assessing response are as follows: (A) Only disease-related ≥Grade 2 organ damage is evaluable as a primary endpoint. (B) Response assessments of CR, PR, SD, PD, and loss of response should only be applied to these ≥Grade 2 organ damage findings in the context of trials. (C) Disease status at the time of patient removal from the study singularly relates to the updated status of initial ≥Grade 2 organ damage finding(s). (D) Exclusion of drug-related toxicity and/or other clinical issues (eg, gastrointestinal tract bleeding in the case of worsening anemia/transfusion-dependence) should be undertaken before assigning the designation PD or loss of response in a patient with worsening of baseline ≥Grade 2 organ damage.

*Responses not maintained for a period of ≥12 weeks do not fulfil criteria for CR, PR, or CI; however, both maintained and unmaintained (<12 weeks duration) responses should be recorded each time they are observed to measure duration of response. [†]Only valid as a response criterion if the pretreatment serum tryptase level is ≥40 ng/mL (ie, if pretreatment serum tryptase is <40 ng/mL, it will not be considered as a criterion in evaluation of response). [‡]Biopsy of organ(s) in addition to the bone marrow to evaluate for SM-related organ damage may be considered. [‖]Preservation of at least 1 CI finding permits a patient to maintain the response of CI if 1 or more CI findings are lost but none meet criteria for PD. However, if 1 or more of the CI findings become PD, then the CI finding assignment is lost and the patient meets criteria for PD. The baseline value for evaluating PD is the pretreatment measurement(s). The PD findings must be considered related to the underlying disease and not to other clinical factors. Progression of an underlying chronic myeloid neoplasm to acute myeloid leukemia is also considered PD.

Supplement Table 2. Non-*KIT* mutations identified in all patients through next-generation sequencing (Tier 1 and Tier 2)

	All Patients (N = 107)
**Mutations in ≥5%, n (%)**	
TET2	69 (64)
SRSF2	38 (36)
GATA2	29 (27)
BCOR	20 (19)
KDM6A	20 (19)
CDKN2A	18 (17)
ASXL1	17 (16)
CUX1	17 (16)
BCORL1	16 (15)
EZH2	16 (15)
IKZF1	13 (12)
RUNX1	13 (12)
CBL	12 (11)
DNMT3A	12 (11)
CEBPA	10 (9)
IDH2	9 (8)
TP53	9 (8)
NRAS	8 (7)
JAK2	7 (7)
SF3B1	6 (6)
U2AF1	6 (6)
KRAS	5 (5)
MPL	5 (5)

Supplement Table 3. Response by mIWG-MRT-ECNM C-findings (response-evaluable population, n = 83)

Presence of evaluable mIWG-MRT-ECNM C-findings	Patients with C-finding at baseline, n (%)*	Patients with clinical improvement in C-findings, n (%)†	Median time to clinical improvement, months
Ascites	15 (18)	8 (53)	4.6
Pleural effusions	11 (13)	6 (55)	3.8
Liver function abnormalities			
Direct bilirubin >1.5 x ULN	9 (11)	1 (11)	12
ALT >3.0 x ULN	0	0	—
AST >3.0 x ULN	0	0	—
ALP >2.5 x ULN	29 (35)	15 (52)	5.5
Hypoalbuminemia	8 (10)	4 (50)	1.2
Splenomegaly	35 (42)	19 (54)	1.9
Neutropenia	3 (4)	0	—
Anemia			
Transfusion-independent	35 (42)	13 (37)	3.8
Transfusion-dependent	4 (5)	1 (25)	5.8
Thrombocytopenia			
Transfusion-independent	16 (19)	3 (19)	14.8
Transfusion-dependent	1 (1)	0	—

ALP, alkaline phosphatase; ALT, alanine aminotransferase; AST, aspartate aminotransferase; mIWG-MRT-ECNM, modified International Working Group-Myeloproliferative Neoplasms Research and Treatment and European Competence Network on Mastocytosis; ULN, upper limit of normal.

*Per response assessment committee adjudication. †Percentages are based on the number of patients with C-finding at baseline.

Supplement Table 4. Response by mIWG-MRT-EC/NM criteria in patients by prior systemic therapy and disease subtype

Best confirmed response, n (%)	Patients with ≥1 prior systemic therapy				Treatment-naïve patients			
		AdvSM subtype				AdvSM subtype		
	All (n = 53)	ASM (n = 8)	SM-AHN (n = 33)	MCL* (n = 12)	All (n = 30)	ASM (n = 5)	SM-AHN (n = 22)	MCL* (n = 3)
ORR[†]<br>95% CI	**35 (66)**<br>**52-79**	6 (75)<br>35-97	22 (67)<br>48-82	7 (58)<br>28-85	**26 (87)**<br>**69-96**	4 (80)<br>28-100	19 (86)<br>65-97	3 (100)<br>29-100
Best response								
CR/CRh	**12 (23)**	3 (38)	7 (21)	2 (17)	**13 (43)**	1 (20)	11 (50)	1 (33)
CR	**6 (11)**	0	4 (12)	2 (17)	**8 (27)**	1 (20)	6 (27)	1 (33)
CRh	**6 (11)**	3 (38)	3 (9)	0	**5 (17)**	0	5 (23)	0
PR	**19 (36)**	3 (38)	11 (33)	5 (42)	**13 (43)**	3 (60)	8 (36)	2 (67)
CI	**4 (8)**	0	4 (12)	0	**0**	0	0	0
SD	**10 (19)**	2 (25)	5 (15)	3 (25)	**3 (10)**	1 (20)	2 (9)	0
PD	**2 (4)**	0	1 (3)	1 (8)	**0**	0	0	0
NE	**6 (11)**	0	5 (15)	1 (8)	**1 (3)**	0	1 (5)	0
TTR, median (range), months	**2.1 (0.5-20.3)**	3.0 (1.8-14.6)	2.0 (0.5-20.3)	2.0 (1.7-12.2)	**3.1 (0.3-15.0)**	1.8 (0.3-15.0)	2.4 (0.5-12.2)	9.2 (9.2-9.3)
Time to CR/CRh, median (range), months	**14.9 (1.8-36.8)**	3.7 (1.8-36.8)	14.8 (1.8-20.3)	23.2 (20.3-26.0)	**9.0 (2.0-25.9)**	25.9 (25.9-25.9)	6.1 (2.0-25.8)	9.3 (9.3-9.3)

95% CI, 95% confidence interval; AdvSM, advanced systemic mastocytosis; CI, clinical improvement; CR, complete remission; CRh, complete remission with partial hematologic recovery; MCL, mast cell leukemia; MCL-AHN, mast cell leukemia with an associated hematologic neoplasm; mIWG-MRT-ECNM, modified International Working Group-Myeloproliferative Neoplasms Research and Treatment and European Competence Network on Mastocytosis; NE, not evaluable; ORR, overall response rate; PR, partial response; PD, progressive disease; SD, stable disease; SM-AHN, systemic mastocytosis with an associated hematologic neoplasm; TTR, time to response.
*The MCL subtype includes patients with the subtypes MCL (n = 11) and MCL-AHN (n = 4). [†]CR + CRh + PR + CI.

Supplement Table 5. Responses by PPR criteria in PPR-evaluable patients by disease subtype

Best confirmed response, n (%)	All (N=107)	AdvSM subtype		
		ASM (n=21)	SM-AHN (n=71)	MCL (n=15)
ORR*<br>95% CI	**79 (74)**<br>**64-82**	17 (81)<br>58-95	52 (73)<br>61-83	10 (67)<br>38-88
Best response				
CR or CRh	**55 (51)**	12 (57)	39 (55)	4 (27)
CR	**33 (31)**	10 (48)	20 (28)	3 (20)
CRh	**22 (21)**	2 (10)	19 (27)	1 (7)
PR	**24 (22)**	5 (24)	13 (18)	6 (40)
SD	**20 (19)**	3 (14)	13 (18)	4 (27)
PD	**0**	0	0	0
NE	**8 (7)**	1 (5)	6 (8)	1 (7)

95% CI, 95% confidence interval; AdvSM, advanced systemic mastocytosis; ASM, aggressive systemic mastocytosis; CR, complete remission; CRh, complete remission with partial hematologic recovery; MCL, mast cell leukemia; NE, not evaluable; ORR, overall response rate; PPR; pure pathologic response; PR, partial response; PD, progressive disease; SD, stable disease; SM-AHN, systemic mastocytosis with an associated hematologic neoplasm.
*CR + CRh + PR.

Supplement Table 6. Patients with disease progression

Patient number	Diagnosis	Age	Avapritinib dose		Baseline disease burden				Best response*	AML progression (yes/no)
			Baseline dose (mg)	Average daily dose (mg)	BM blasts (%)	BM MC %)	Serum tryptase (µg/L)	*KIT* D816V in PB (%)		
1	ASM	64	200	110	1	75	226	11.6	PR	N
2	MCL	69	200	93	2	90	382	15.77	SD	N
3	MCL-CMML-1	67	200	111	5	80	728	0.06	SD	Y
4	MCL-MDS/MPN-U	37	200	240	1	80	1600	0.02	PD	N
5	SM-CEL	76	200	84	4	20	464	38.41	PR	N
6	SM-CMML-0	71	200	98	1	15	488	44	PR	Y
7	SM-CMML-0	79	200	50	2	20	1116	3.85	CRh	N
8	SM-CMML-0	78	200	103	2	20	49	2.7	PR	Y
9	SM-CMML-0	69	200	48	1	75	604	36.26	SD	Y
10	SM-CMML-0	63	200	74	1	40	107	2.88	PR	N
11	SM-CMML-0	85	200	59	1	80	107	25.36	PD	N
12	SM-CMML-0	74	200	100	1	30	508	39.88	PR	N
13	SM-CMML-1	47	200	200	5	1	47	42.86	PD	Y
14	SM-CMML-1	54	200	86	4	30	37	0.13	CRh	Y
15	SM-CMML-1	73	200	65	7	10	87	16.4	PR	N
16	SM-MDS/MPN-U	69	200	73	1	70	116	41.75	SD	N
17	SM-MDS/MPN-U	79	200	65	1	90	160	18.72	PR	N
18	SM-MDS-MLD	65	200	200	2	15	122	0.01	SD	N
19	SM-MDS-RS-MLD	77	200	200	1	30	568	47.76	PD	N
20	SM-MPN	72	200	200	3	20	176	6	PR	N
21	SM-MPN	64	200	200	3	15	312	21.92	PD	N

AML, acute myeloid leukemia; ASM, aggressive systemic mastocytosis; BM, bone marrow; CEL, chronic eosinophilic leukemia; CMML, Chronic Myelomonocytic Leukemia; CRh, complete remission with partial hematologic recovery; MC, mast cell; MCL mast cell leukemia; MDS, myelodysplastic syndrome; MLD, multilineage dysplasia; MPN, myeloproliferative neoplasm; MDS/MPN-U, myelodysplastic/myeloproliferative neoplasm, unclassifiable; PB, peripheral blood; PD, progressive disease; PR, partial response; RS, ring sideroblasts; SD, stable disease; SM, systemic mastocytosis.
*Per modified International Working Group-Myeloproliferative Neoplasms Research and Treatment and European Competence Network on Mastocytosis criteria.

Supplement Table 7. SAEs and treatment-related SAEs of safety population (N = 107)

	Any-cause SAEs	Treatment-related SAEs
	Any grade	Any grade
**Any, n (%)**	69 (64)	15 (14)
**Non-hematological SAEs in ≥2%, n (%)**		
COVID-19	4 (4)	0
Gastrointestinal hemorrhage	4 (4)	1 (<1)
Nephrolithiasis	4 (4)	0
Pneumonia	4 (4)	0
Acute kidney injury	3 (3)	1 <1)
Appendicitis	3 (3)	0
Cardiac failure	3 (3)	0
Chronic kidney disease	3 (3)	1 (<1)
COVID-19 pneumonia	3 (3)	0
Diverticulitis	3 (3)	1 (<1)
Ascites	2 (2)	0
Cellulitis	2 (2)	0
Colon cancer	2 (2)	0
Disease progression	2 (2)	0
Diverticulitis (perforated intestinal)	2 (2)	0
Dysphagia	2 (2)	0
Erysipelas	2 (2)	0
Gastroenteritis	2 (2)	0
Infection	2 (2)	0
Intra-abdominal hemorrhage	2 (2)	0
Peritonitis	2 (2)	0
Procedural hemorrhage	2 (2)	1 (<1)
Pyrexia	2 (2)	0
Renal failure	2 (2)	0
Respiratory tract infection	2 (2)	0
Sepsis	2 (2)	0
Small intestinal obstruction	2 (2)	0
Transient ischemic attack	2 (2)	0
Upper limb fracture	2 (2)	0
Urinary tract infection	2 (2)	0
**Hematological SAEs in ≥2%, n (%)**		
Anemia*	4 (4)	2 (2)
Thrombocytopenia†	2 (2)	2 (2)
**AEs of special interest in ≥2%, n (%)**		
**Cognitive effect**		
Cognitive disorder	4 (4)	3 (3)

**Intracranial bleeding**		
Intracranial hemorrhage	2 (2)	2 (2)
Subdural hematoma	2 (2)	2 (2)

AE, adverse event; SAE, serious adverse event.
*Grouped term that includes anemia and hemoglobin decreased. †Grouped term that includes thrombocytopenia and platelet count decreased.

Supplement Table 8. Median time to onset, time to improvement, and time to resolution of cognitive effects

	All (N = 107)
**Time to onset of any Grade cognitive effects**	
Patients with ≥1 event, n (%)	36 (34)
Time to onset, median (range), weeks	25.9 (0.1-216.6)
**Time to improvement of Grade ≥2 cognitive effects**	
Patients with ≥1 event, n (%)	13 (12)
Time to improvement, median (range), weeks	8.0 (0.3-88.0)
**Time to resolution of Grade ≥2 cognitive effects**	
Patients with ≥1 event, n (%)	11 (10)
Time to resolution, median (range), weeks	9.1 (0.3-125.4)

Supplement Table 9. Patient baseline characteristics, avapritinib dosing, platelet count, and timing of the occurrence of intracranial bleeding events

Age (years)	Gender	Starting daily dose (mg; dose prior to event)	RAC-adjudicated diagnosis	Adverse event/severity grade	Day of occurrence	Platelet count* baseline/prior to event visit (×10^9/L)	Confounding factors/comments
59	Female	200 (100)	SM-AHN	Subdural hematoma Grade 2	85	49/33	Medical history of SM related thrombocytopenia, Grade 1 INR increased, CML, and alcohol abuse. Patient received 1 dose of enoxaparin 2 days prior to Grade 2 ICB for DVT prophylaxis.
		(200)		Subdural hematoma Grade 4	243	49/167	
79	Male	200 mg (50 mg)	SM-AHN	Subdural hematoma Grade 3	88	141/101	Medical history of Grade 1 ongoing thrombocytopenia and Grade 2 hypertension. Patient had several episodes of Grade 3 thrombocytopenia leading to dose reductions. Head trauma with loss of consciousness around the time of the event.
72	Male	200 mg (50/100 mg alternative days)	SM-AHN	Intracranial hemorrhage Grade 2	991	57/101	Medical history of hypertension, hypercholesterolemia, and SM related thrombocytopenia. Patient received prohylactic dose–dalteparin during inpatient hospitalization starting ~10 days prior to ICB.<br><br>Patient had several dose reductions due to AEs including thrombocytopenia (received eltrombopag) and dose escalation. Patient had platelet count trending between 50-100×10^9/L most of the time during the study.
68	Female	200 (200)	ASM	Intracranial hemorrhage Grade 1	400	207/129	Medical history of hypertension and had Grade 3 hypertension around the time of ICB. Concomitant medication included treatment with tinzaparin sodium 2 days prior to ICB which continued 10 days after.

AE, adverse event; ASM, aggressive systemic mastocytosis; CML, chronic myeloid leukemia; DVT, deep vein thrombosis; ICB, intracranial bleed; INR, international normalized ratio; RAC, response assessment committee; SM, systemic mastocytosis; SM-AHN, systemic mastocytosis with an associated hematological neoplasm.

Supplement Table 10. TEAEs leading to dose modification

	All doses (N = 107)	
	Any grade	Grade ≥3
**Any TEAE leading to dose interruption, n (%)**	78 (73)	71 (66)
**TEAEs leading to dose interruption in ≥5%, n (%)**		
Thrombocytopenia*,†	27 (25)	26 (24)
Neutropenia‡	24 (22)	24 (22)
Anemia‖	11 (10)	10 (9)
Cognitive disorder	11 (10)	3 (3)
White blood cell count decreased	7 (7)	5 (5)
Periorbital edema	5 (5)	5 (5)
**Any TEAE leading to dose reduction, n (%)**	85 (79)	56 (52)
**TEAEs leading to dose reduction in ≥5%, n (%)**		
Thrombocytopenia*,†	32 (30)	25 (23)
Neutropenia‡	23 (21)	22 (21)
Cognitive disorder	12 (11)	2 (2)
Periorbital edema#	12 (11)	4 (4)
Peripheral edema¶	11 (10)	1 (<1)
Anemia‖	5 (5)	2 (2)
**Any TEAE leading to treatment discontinuation, n (%)**	38 (36)	29 (27)
**TEAEs leading to treatment discontinuation in ≥2%, n (%)**		
Acute kidney injury	3 (3)	3 (3)
Anemia‖	3 (3)	2 (2)
Cognitive disorder	3 (3)	2 (2)
Neutropenia‡	3 (3)	3 (3)
Thrombocytopenia*,†	3 (3)	3 (3)
Intracranial hemorrhage	2 (2)	0
Subdural hematoma	2 (2)	2 (2)

TEAE, treatment-emergent adverse event.
*Grouped term that includes thrombocytopenia and platelet count decreased. †Per the study protocol, patients who had a platelet count ≤50x10^9/L were required to undergo dose interruption and dose reduction until platelet counts were ≥50x10^9/L for 4 weeks. ‡Grouped term that includes neutropenia and neutrophil count decreased. ‖Grouped term that includes anemia and hemoglobin decreased. ¶Grouped term that includes peripheral edema and peripheral swelling. #Grouped term that includes eyelid edema, periorbital edema, periorbital swelling, and swelling of eyelid.

Supplement Table 11. TRAEs leading to dose modification

	All doses (N = 107)	
	Any grade	Grade ≥3
**Any TRAE leading to dose interruption, n (%)**	69 (64)	62 (58)
**TRAEs leading to dose interruption in ≥5%, n (%)**		
Thrombocytopenia*,†	27 (25)	26 (24)
Neutropenia‡	22 (21)	22 (21)
Cognitive disorder	10 (9)	3 (3)
Anemia‖	8 (7)	7 (7)
White blood cell count decreased	7 (7)	5 (5)
Periorbital edema	5 (5)	5 (5)
**Any TRAE leading to dose reduction, n (%)**	83 (78)	55 (51)
**TRAEs leading to dose reduction in ≥5%, n (%)**		
Thrombocytopenia*,†	32 (30)	25 (23)
Neutropenia‡	22 (21)	21 (20)
Periorbital edema#	12 (11)	4 (4)
Cognitive disorder	11 (10)	2 (2)
Peripheral edema¶	11 (10)	1 (<1)
Anemia‖	5 (5)	2 (2)
**Any TRAE leading to treatment discontinuation, n (%)**	20 (19)	12 (11)
**TRAEs leading to treatment discontinuation in ≥1%, n (%)**		
Cognitive disorder	3 (3)	2 (2)
Anemia‖	2 (2)	1 (<1)
Intracranial hemorrhage	2 (2)	0
Neutropenia	2 (2)	2 (2)
Subdural hematoma	2 (2)	2 (2)
Thrombocytopenia*,†	2 (2)	2 (2)

TRAE, treatment-related adverse event.
*Grouped term that includes thrombocytopenia and platelet count decreased. †Per the study protocol, patients who had a platelet count ≤50x10^9/L were required to undergo dose interruption and dose reduction until platelet counts were ≥50x10^9/L for 4 weeks. ‡Grouped term that includes neutropenia and neutrophil count decreased. ‖Grouped term that includes anemia and hemoglobin decreased. ¶Grouped term that includes peripheral edema and peripheral swelling. #Grouped term that includes eyelid edema, periorbital edema, periorbital swelling, and swelling of eyelid.

Supplement Table 12. Median dosing details

	All patients (N = 107)	AdvSM subtype			Patients with ≥1 prior systemic therapy (n = 69)	Treatment-naïve patients (n = 38)
		ASM (n = 21)	SM-AHN (n = 71)	MCL (n = 15)		
Average daily dose, median* (range), mg	106 (27-240)	119 (30-200)	101 (27-200)	129 (44-240)	110 (27-240)	101 (37-200)
Time to first dose interruption,† median (range), months	1.8 (0.2-32.4)	3.2 (0.3-22.5)	1.2 (0.2-32.4)	2.2 (0.3-17.7)	1.8 (0.2-29.0)	1.6 (0.3-32.4)
Time to first dose reduction,† median (range), months	1.6 (0.0-34.2)	3.1 (0.6-34.2)	1.5 (0.0-31.1)	1.7 (0.1-20.3)	1.6 (0.0-34.2)	1.7 (0.6-31.1)

AdvSM, advanced systemic mastocytosis; ASM, aggressive systemic mastocytosis; MCL, mast cell leukemia; SM-AHN, systemic mastocytosis with an associated hematological neoplasm.
*Average daily dose over duration of treatment was determined for each individual patient; data shown here are medians of the average daily doses across all patients. †Individual patients could have dose interruptions and dose reductions due to the same AE.

Supplement Table 13. Dosing summary

	All patients, n (%)					
	200 mg	150 mg	100 mg	75 mg	50 mg	25 mg
**Dose by timepoint**						
6 months (n = 86)	21 (24)	0	44 (52)	0	16 (19)	5 (6)
12 months (n = 78)	16 (21)	1 (1)	41 (53)	1 (1)	13 (17)	6 (8)
18 months (n = 69)	13 (19)	1 (1)	33 (48)	1 (1)	13 (19)	8 (12)
24 months (n = 60)	9 (15)	2 (3)	29 (48)	1 (2)	12 (20)	7 (12)
30 months (n = 54)	9 (17)	2 (4)	26 (48)	1 (2)	9 (17)	7 (13)
36 months (n = 49)	6 (12)	2 (4)	23 (47)	1 (2)	12 (24)	5 (10)
42 months (n = 43)	4 (9)	1 (2)	21 (49)	1 (2)	11 (26)	4 (9)
48 months (n = 29)	2 (7)	1 (3)	12 (41)	0	12 (41)	2 (7)
54 months (n = 15)	1 (7)	1 (7)	5 (33)	0	7 (47)	1 (7)
60 months (n = 4)	0	0	2 (50)	0	2 (50)	0

Supplemental Table 14. Univariate and multivariate logistic regression analyses of predictors of response in response-evaluable patients (mIWG-MRT-ENCM criteria)

	Univariate		Multivariate	
**Effect**	**OR (95% CI)**	***P***	**OR (95% CI)**	***P***
ASM vs MCL diagnosis	1.67 (0.31-8.93)	.55	0.47 (0.05-4.15)	.50
SM-AHN vs MCL diagnosis	1.16 (0.34-3.93)	.82	4.78 (0.31-74.30)	.26
Presence of an AHN (no/yes)*	1.90 (0.62-5.87)	.27	8.71 (0.43-175.61)	.16
Baseline *KIT* D816V VAF	1.00 (0.98-1.03)	.81	1.01 (0.97-1.05)	.61
S/A/R mutation status (negative/positive)	1.79 (0.68-4.71)	.24	3.19 (0.86-11.88)	.08
Baseline serum tryptase	1.00 (1.00-1.00)	.46	1.00 (1.00-1.00)	.74
Baseline BM mast cell burden	1.00 (0.98-1.02)	.75	1.00 (0.98-1.02)	.87
Previous antineoplastic therapy (no/yes)	4.19 (1.27-13.83)	.02	5.68 (1.46-22.09)	.01

AHN, associated hematological neoplasm; ASM, aggressive systemic mastocytosis; BM, bone marrow; MCL, mast cell leukemia; mIWG-MRT-ECNM, modified International Working Group-Myeloproliferative Neoplasms Research and Treatment and European Competence Network on Mastocytosis; OR, odds ratio for likelihood of response; *S/A/R*, *SRSF2*, *ASXL1*, and/or *RUNX1*; SM-AHN, systemic mastocytosis with an associated hematological neoplasm; VAF, variant allele frequency.
*Included both SM-AHN and MCL-AHN.

Supplement Table 15. Multivariate Cox regression analysis of predictors of OS

	Univariate		Multivariate	
**Effect**	**HR (95% CI)**	***P***	**HR (95% CI)**	***P***
Age	1.02 (0.98-1.07)	.29	1.08 (1.02-1.14)	.008
ASM vs MCL diagnosis	0.23 (0.03-1.99)	.18	0.08 (0.01-1.36)	.08
SM-AHN vs MCL diagnosis	1.17 (0.44-3.10)	.76	0.51 (0.08-3.19)	.47
Presence of an AHN (no/yes)*	0.23 (0.07-0.75)	.01	1.15 (0.12-10.70)	.90
Best *KIT* D816V status (negative/positive)	1.04 (0.36-3.05)	.94	3.02 (0.57-16.02	.19
S/A/R mutation status (negative/positive)	0.47 (0.21-1.03)	.06	0.92 (0.34-2.53)	.87
Baseline serum tryptase	1.00 (1.00-1.00)	.12	1.00 (1.00-1.00)	.03
Baseline BM mast cell burden	0.99 (0.98-1.01)	.17	0.98 (0.96-1.00)	.01
Baseline *KIT* D816V VAF	0.98 (0.95-1.00)	.08	0.96 (0.93-0.99)	.13
Previous antineoplastic therapy (no/yes)	0.35 (0.13-0.92)	.03	0.25 (0.07-0.95)	.04
CR/CRh or PR (yes/no)	0.12 (0.05-0.28)	< .0001	0.09 (0.03-0.26)	< .0001
CR/CRh or PR or CI (yes/no)†	0.12 (0.06-0.28)	< .0001	—	—

95% CI, 95% confidence interval; AHN, associated hematologic neoplasm; ASM, aggressive systemic mastocytosis; BM, bone marrow; CI, clinical improvement; CR, complete remission; CRh, complete remission with partial hematologic recovery; HR, hazard ratio for likelihood of death; MCL, mast cell leukemia; MCL-AHN, mast cell leukemia with an associated hematologic neoplasm; mIWG-MRT-ECNM, modified International Working Group-Myeloproliferative Neoplasms Research and Treatment and European Competence Network on Mastocytosis; OS, overall survival; PR, partial remission; S/A/R, *SRSF2, ASXL1*, and/or *RUNX1*; SM-AHN, systemic mastocytosis with an associated hematological neoplasm; VAF, variant allele frequency. *Included both SM-AHN and MCL-AHN. †CR/CRh or PR or CI was not included as a parameter in multivariate analyses of OS.

Supplement Figure 1. Analysis populations (all patients)

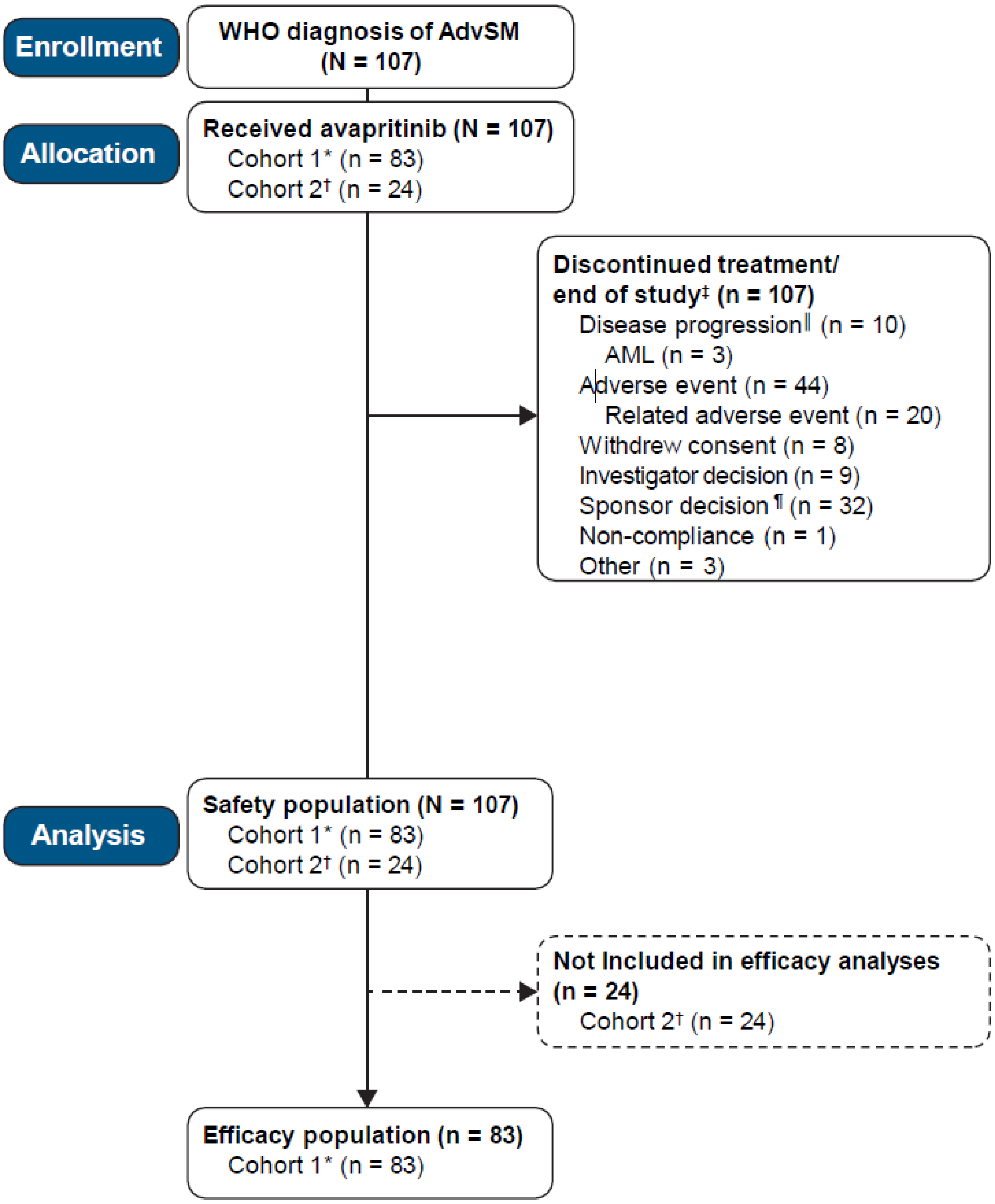

AdvSM, advanced systemic mastocytosis; AML, acute myeloid leukemia; ASM, aggressive systemic mastocytosis; MCL, mast cell leukemia; mIWG-MRT-ECNM, modified International Working Group-Myeloproliferative Neoplasms Research and Treatment and European Competence Network on Mastocytosis; OS, overall survival; SM-AHN, systemic mastocytosis with an associated hematological neoplasm; SSC, study steering committee; WHO, World Health Organization.
In total, 2 patients had a starting dose of 100 mg once a day. A dose of 100 mg was selected for these patients as they had platelet counts between $25x10^9$ and $50x10^9$/L and were enrolled prior to the protocol amendment that excluded patients with platelet counts <$50x10^9$/L.
*Cohort 1: Patients with AdvSM and ≥1 mIWG-MRT-ECNM criteria for evaluable disease (have severe and quantifiable organ damage [an evaluable C-finding] or have MCL [regardless of C-findings]) as confirmed by the SSC.
†Cohort 2: Patients with AdvSM who were not considered eligible for an adjudicated response and were confirmed centrally to have ASM or SM-AHN but were lacking an evaluable C-finding as determined by the SSC.
‡No treatment discontinuations were due to lost to follow-up, protocol deviation, pregnancy, or death.
‖Disease progressions listed here are locally reported primary reasons for treatment discontinuations and do not reflect the 21 post-hoc centrally-evaluated disease progressions described within the manuscript.
¶Sponsor decision' represents patients still receiving avapritinib at end of study; all patients continued to receive avapritinib post study from either commercial supply (n=31) or in a roll-over study (n=1).

Supplement Figure 2. mIWG-MRT-ECNM responses over time

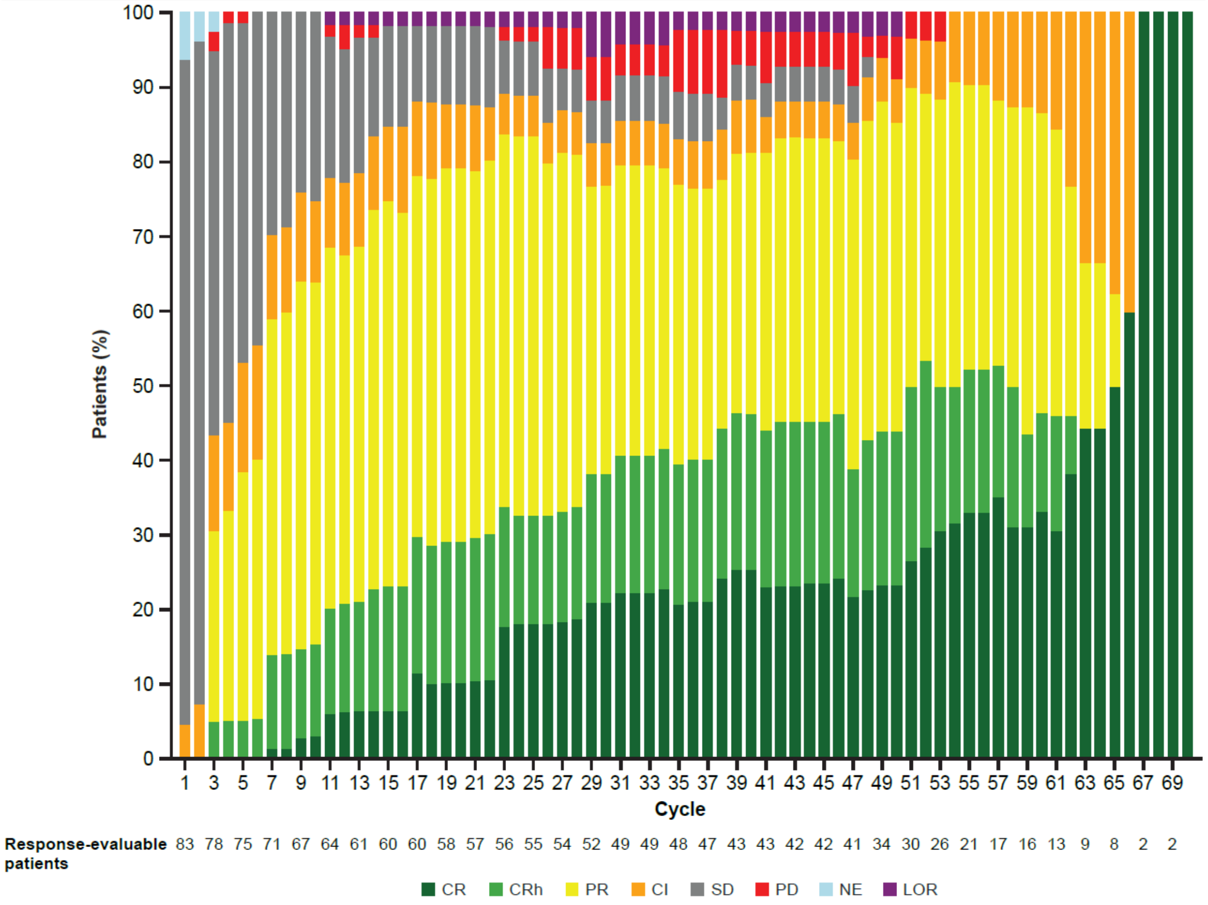

CI, clinical improvement; CR, complete remission; CRh, complete remission with partial hematologic recovery; LOR, loss of response; mIWG-MRT-ECNM, modified International Working Group-Myeloproliferative Neoplasms Research and Treatment and European Competence Network on Mastocytosis; NE, not evaluable; PR, partial response; PD, progressive disease; SD, stable disease.

Supplement Figure 3. Duration of response in all response-evaluable patients by AdvSM subtype and prior treatment history. (A) All response-evaluable patients. (B) Patients with at least 1 prior systemic therapy. (C) Treatment-naïve patients.

A

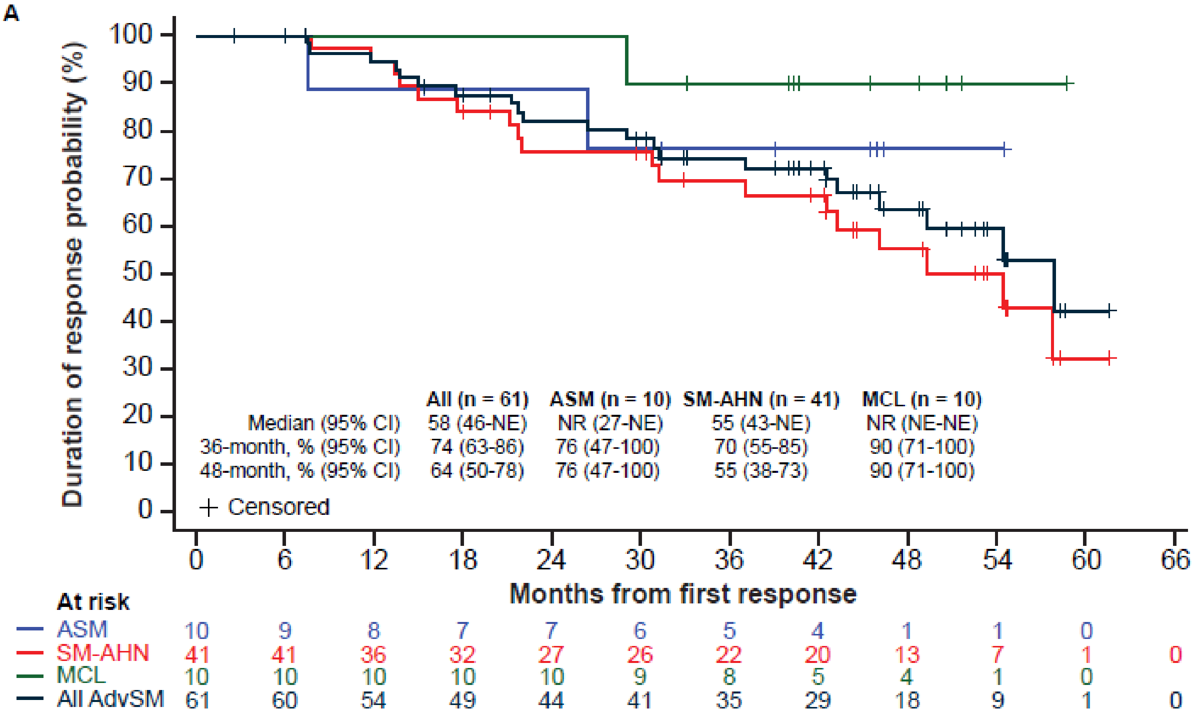

B

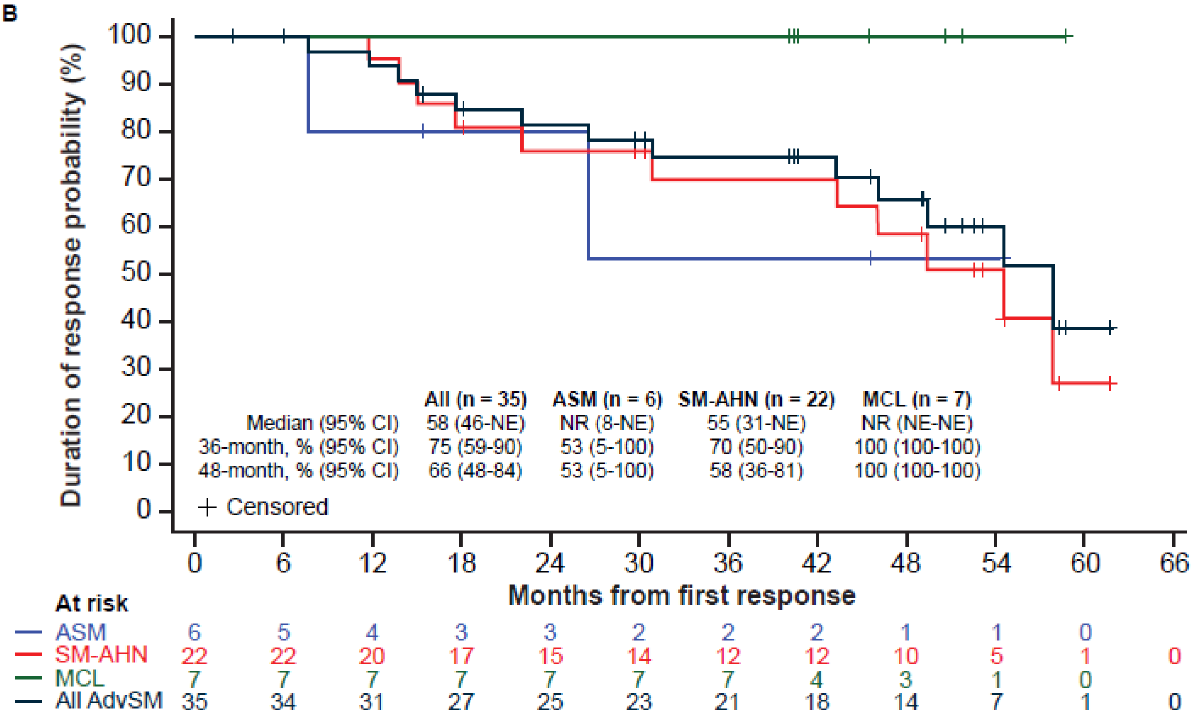

C

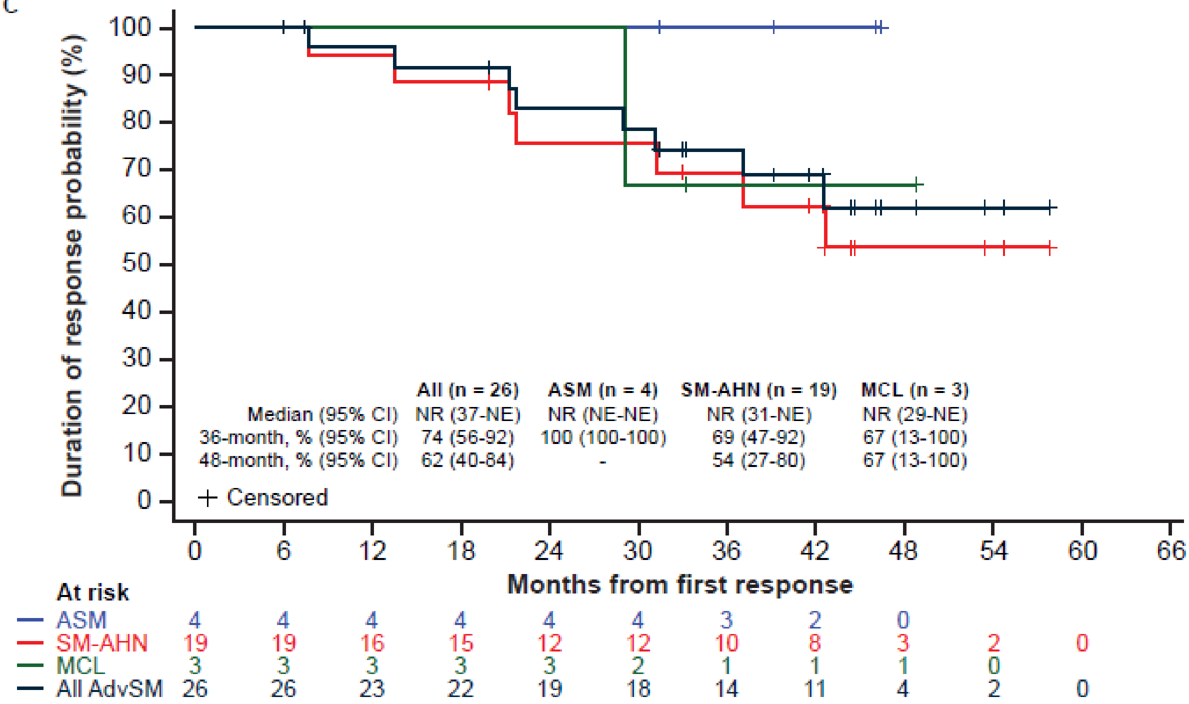

AdvSM, advanced systemic mastocytosis; ASM, aggressive systemic mastocytosis; MCL, mast cell leukemia; NR, not reached; SM-AHN, systemic mastocytosis with an associated hematological neoplasm.

Supplement Figure 4. Progression-free survival in response-evaluable patients by AdvSM subtype and prior treatment history. (A) Patients with at least 1 prior systemic therapy. (B) Treatment-naïve patients.

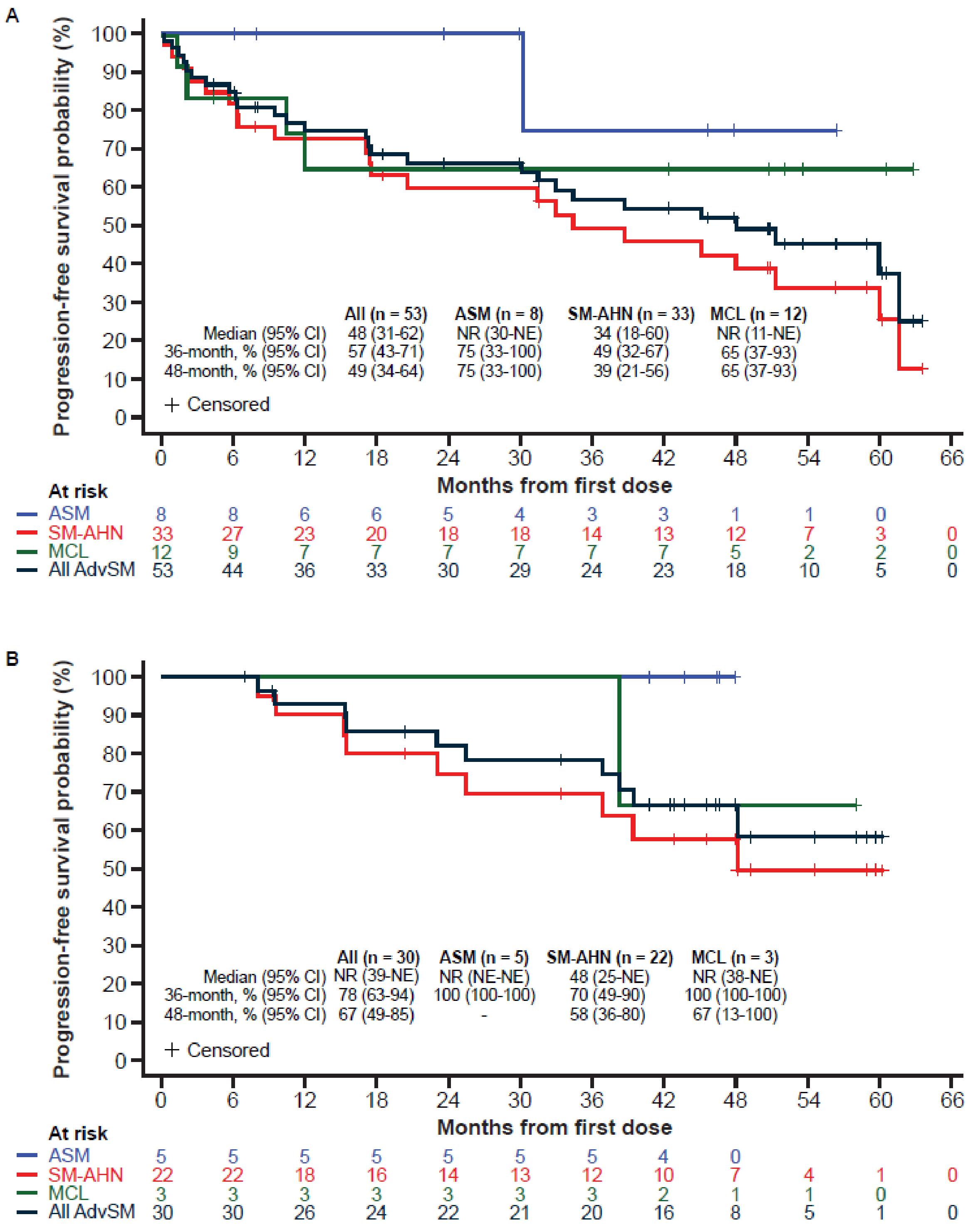

AdvSM, advanced systemic mastocytosis; ASM, aggressive systemic mastocytosis; MCL, mast cell leukemia; NR, not reached; SM-AHN, systemic mastocytosis with an associated hematological neoplasm.

Supplement Figure 5. Overall survival in safety population by AdvSM subtype and prior treatment history. (A) Patients with at least 1 prior systemic therapy. (B) Treatment-naïve patients.

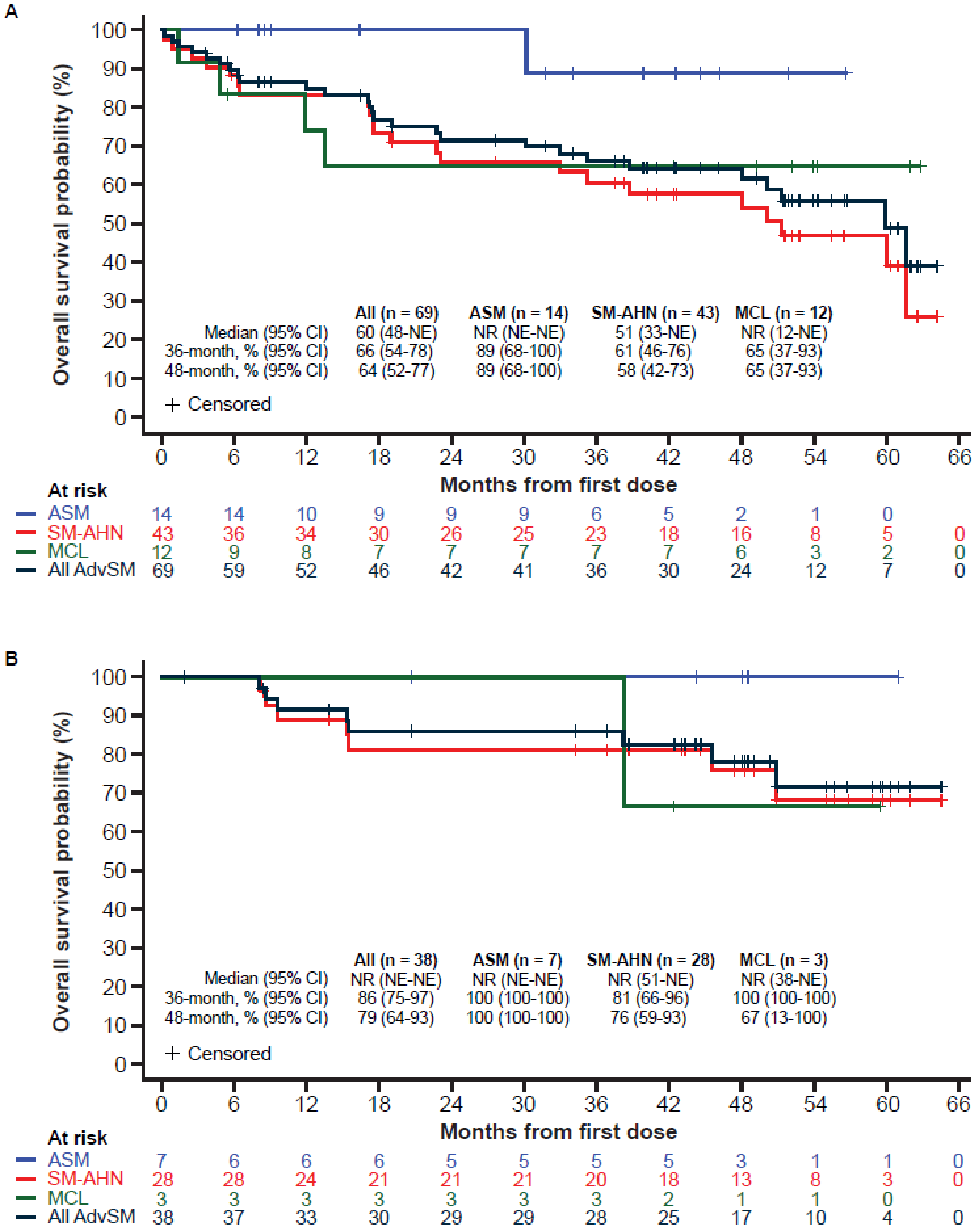

AdvSM, advanced systemic mastocytosis; ASM, aggressive systemic mastocytosis; MCL, mast cell leukemia; NR, not reached; SM-AHN, systemic mastocytosis with an associated hematological neoplasm.

Supplement Figure 6. OS in patients with AdvSM by number of C-findings at baseline

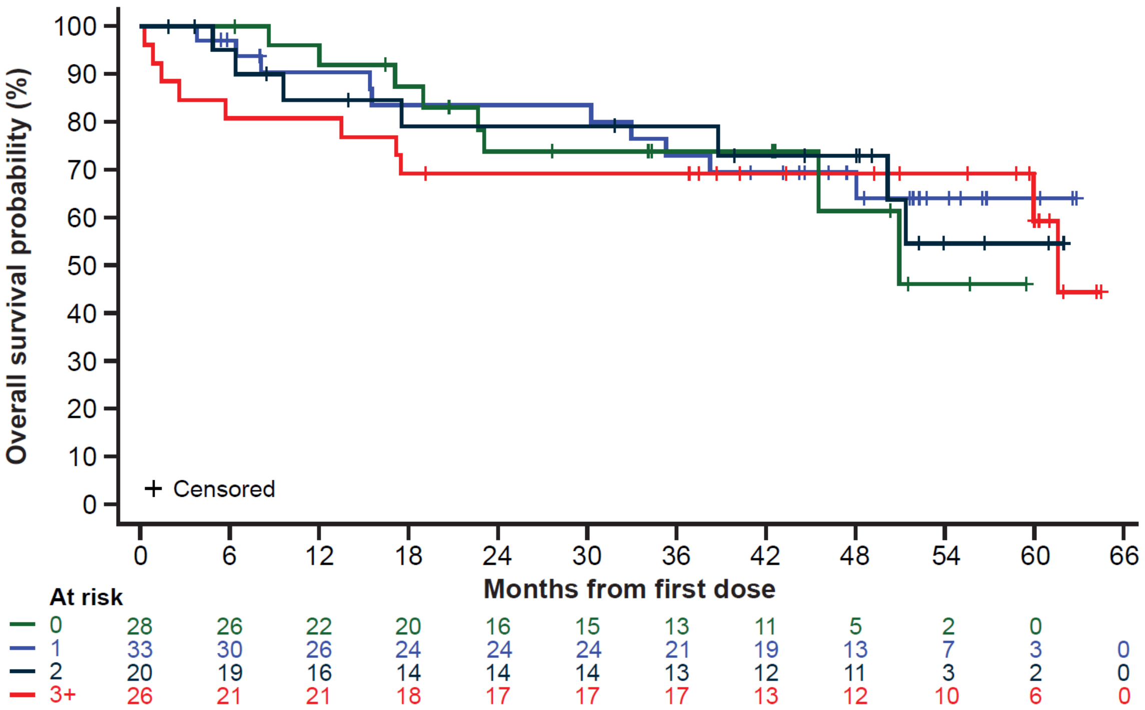

OS, overall survival.

Supplement Figure 7. OS by MARS.

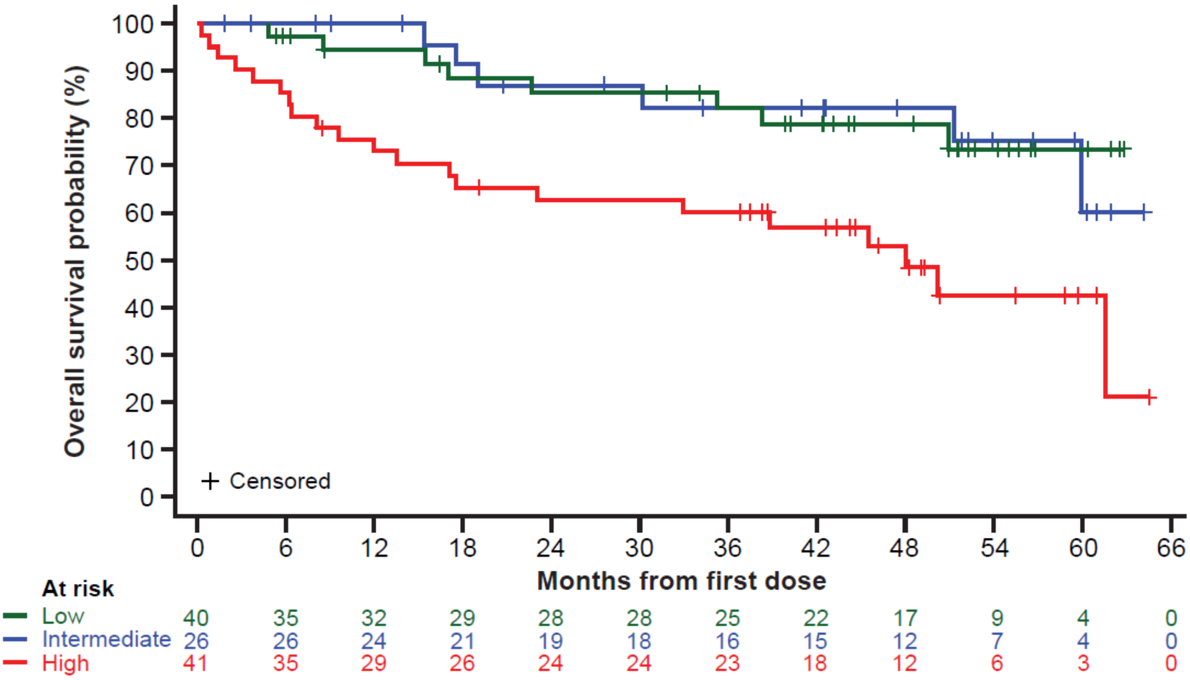

MARS, Mutation-Adjusted Risk Score; OS, overall survival.
